# Well-Defined Cu Precatalysts Indicate Design Rules for Reactivity in Nitrate Electroreduction

**DOI:** 10.1021/jacs.5c09246

**Published:** 2025-09-22

**Authors:** Jia Du, Anna Loiudice, Krishna Kumar, Ludovic Zaza, Raffaella Buonsanti

**Affiliations:** Laboratory of Nanochemistry for Energy (LNCE), Institute of Chemical Sciences and Engineering (ISIC), 150727 École Polytechnique Fédérale de Lausanne, CH-1950 Sion, Switzerland

## Abstract

The electrochemical nitrate reduction reaction (NO_3_RR) to ammonia (NH_3_) is a promising route for sustainable NH_3_ synthesis. Cu-based materials are the most used and promising catalysts for this reaction. However, Cu undergoes uncontrollable compositional and structural changes under NO_3_RR conditions, necessitating a deeper understanding of the relationship between precatalyst features, structural evolution, and catalytic performance for the advancement of current catalyst design rules. Here, we exploit well-defined Cu and Cu oxide nanocrystals (NCs) as precatalysts to elucidate these correlations. We find that the size, shape, and oxide content of the Cu precatalysts all play a role in driving structural evolution and, thus, the catalytic behavior during NO_3_RR. In particular, a higher oxide content, an optimized {111}/{100} facet ratio, and the spatial proximity of these facets forming grain boundaries within the active catalysts emerge as key factors to enhance NH_3_ selectivity. Among the studied Cu precatalysts, 10 nm Cu spheres integrate these key features, achieving a competitive NH_3_ production rate compared to the state of the art. This work links pre- and in situ-formed catalyst features to catalytic performance, offering insights into the morphological dynamics of Cu catalysts under NO_3_RR conditions.

## Graphical abstract

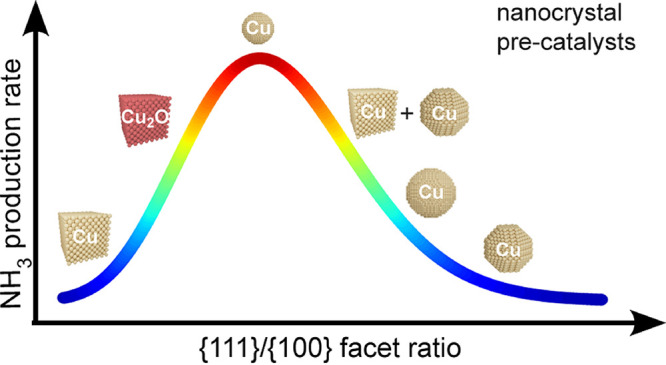

## Introduction

Ammonia (NH_3_) is a crucial chemical in different areas, including agriculture, the chemical industry, the energy sector, and food production.^1^ The current industrial method for NH_3_ production is the Haber-Bosch process, which is highly energetically demanding because of the high temperature and pressure needed for nitrogen (N_2_) and hydrogen (H_2_) to react.^2–5^

The electrochemical nitrate reduction reaction (NO_3_RR) is a promising reaction for NH_3_ production at atmospheric pressure and temperature.^6,7^ Furthermore, the NO_3_RR provides a means to utilize nitrate pollutants.^6,7^ As such, this reaction currently attracts significant interest at both fundamental and applied levels.

The electrochemical conversion of nitrate (NO_3_^–^) to NH_3_ occurs via two primary steps, wherein the reduction of NO_3_^–^ to NO_2_^–^ is followed by the reduction of NO_2_^–^ to NH_3_.^8,9^ Cu-based materials are the most studied catalysts for NO_3_RR, as Cu has a favorable adsorption energy for NO_3_^–^, which is beneficial for the reaction to occur.^10^

However, the fundamental understanding of the compositional and structural characteristics responsible for the tuning of NH_3_ selectivity and production rate in the NO_3_RR on Cu itself remains elusive.

Studies on Cu single crystals have evidenced structural sensitivity, with the {100} surface enhancing NO_2_^–^ adsorption compared to the {111} surface in alkaline media.^11^ One study corroborated this observation using wet-etched Cu foil modified with Cu nanocubes, exposing mostly the {100} facets and showing enhanced NO_3_RR activity and NH_3_ selectivity in a KOH-based electrolyte.^12^ At the same time, several studies exist which suggest that {111} facets exhibit greater NH_3_ productivity compared to {100}.^13,14^ For instance, density functional theory indicated superior NO_3_RR performance on {111} than on {100} in near-neutral and basic media.^13^ Additionally, one study on Cu nanosheets, predominantly exposing {111} facets, reported higher NH_3_ Faradaic efficiency and production rate than Cu nanocubes, predominantly exposing {100} facets, under the same conditions, indicating enhanced selectivity toward NH_3_ over NO_2_^–^ on {111} facets.^14^

The discrepancies observed in these studies can be partially attributed to variations in experimental conditions, such as electrolyte composition, pH, and so forth.^11–14^ Additionally, dynamic structural and compositional changes of Cu under cathodic potential might occur and, thus, impact NO_3_RR performance.^15–20^ For example, Cu_2_O cubes were reported to reconstruct under NO_3_RR operational conditions, which results from the reduction of Cu oxides to metallic Cu.^15^ Similarly, CuO nanosheets were reported to undergo conversion to metallic Cu, accompanied by lattice rearrangement that eventually formed {100} and {111} facets in the post-NO_3_RR sample.^18^ Consequently, correlating NO_3_RR performance to the composition and structure of the initial Cu material used as the catalyst can lead to misinterpretation.

Generally, an open question exists regarding the relationship between the composition (i.e., copper oxide, metallic copper) and structural features of the initial catalytic material (i.e., the precatalyst) and its evolution under operating conditions in the NO_3_RR. Unveiling this relationship is essential to advance the current design rules of catalysts for the NO_3_RR.

Herein, we exploit well-defined Cu and Cu oxide nanocrystals (NCs) with tunable shape (i.e., exposed surfaces) and size to establish a correlation between the structure and composition of the precatalysts, their restructuring under NO_3_RR conditions and, in turn, their NH_3_ selectivity and production rate. We find that all Cu NCs reconstruct, although to different extents, which depend on the initial oxidation state, exposed facets, and particle size. We determine that the highest NH_3_ selectivity and production rate require the simultaneous presence of a high oxide fraction and the coexistence of {100} and {111} facets in the precatalysts. More specifically, we evidence that an optimal ratio and spatial proximity of these facets in the reconstructed active catalysts are crucial to achieving optimal performance.

## Results and Discussion

### Characterizations of the Studied Cu NCs

Cu NCs with octahedral, cubic, and spherical morphologies were first studied as catalytic materials for NO_3_RR. Cu octahedra (denoted as Cu-Octa) and Cu cubes (denoted as Cu-Cube) were selected due to the predominant exposure of {111} and {100} facets, respectively. Cu spheres were included to investigate the mixed {111}/{100} facet behavior. Cu spheres with average diameters of 10 nm (Cu–S10) and 20 nm (Cu–S20) were used to study the size effect. These NCs were prepared using colloidal methods (Supporting Information).^21–24^

Figure 1a,b reports the morphological and structural characterization of the as-prepared Cu NCs. Bright-field transmission electron microscopy (TEM) images and the corresponding statistical analysis confirm the high uniformity of the as-synthesized Cu NCs in both size and shape (Figures 1a and S1). Grazing incidence X-ray diffraction (GI-XRD) patterns show the characteristic diffraction planes corresponding to metallic Cu{111} and Cu{100} facets for all samples and are consistent with the predominantly exposed facets for each sample (Figure 1b). All as-synthesized samples exhibit a certain fraction of Cu_2_O, which results from their self-passivation upon exposure to air. The higher intensity of the Cu_2_O diffraction peak and, thus, the fraction of Cu_2_O for the 10 nm spheres is consistent with their higher surface-to-volume ratio.^25^

**Figure 1:**
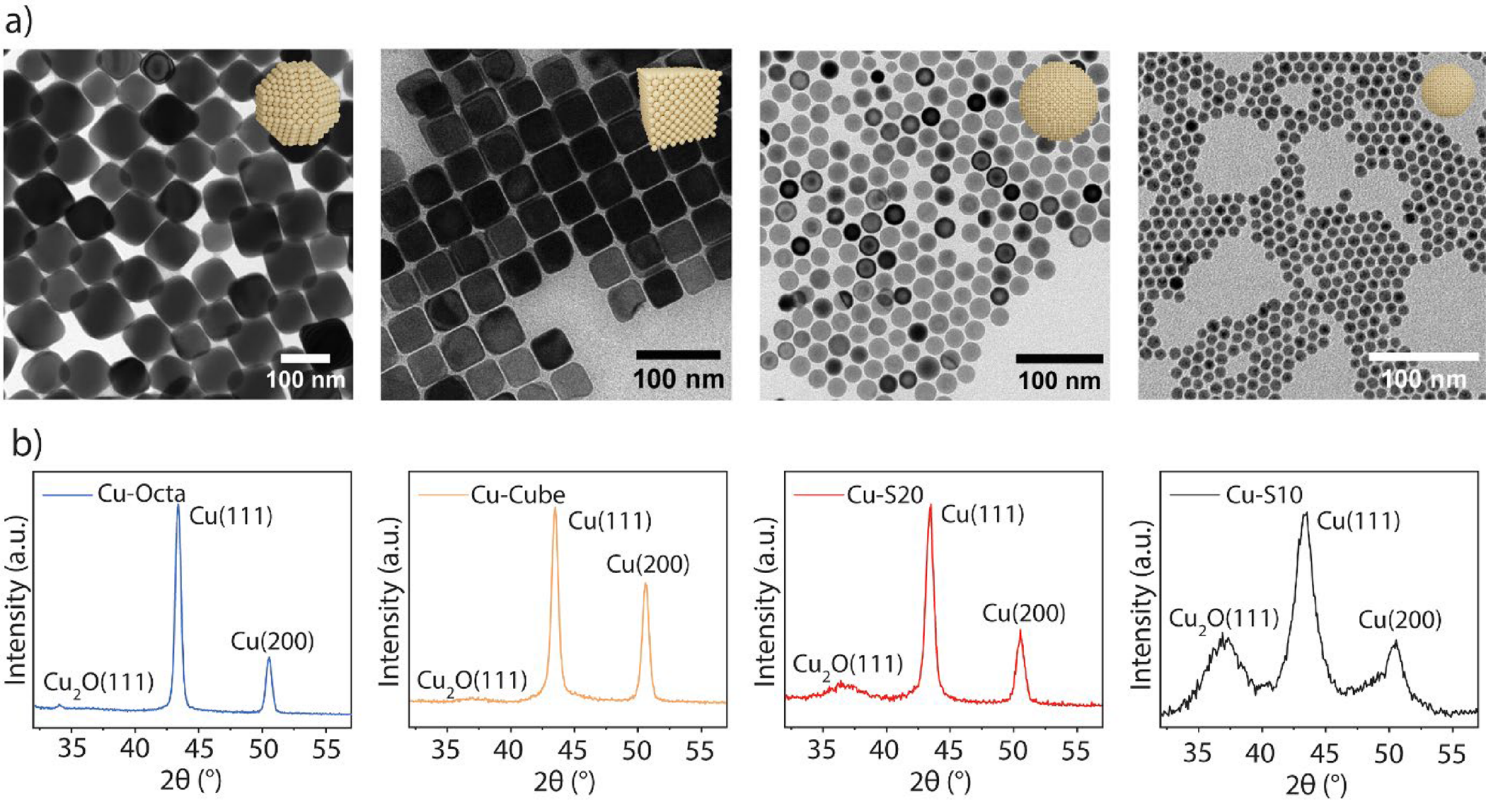
Characterizations of the as-synthesized Cu NCs. (a) Bright-field TEM images of as-synthesized Cu NCs: Cu-Octa, Cu-Cube, Cu–S20, and Cu–S10 from left to right; (b) GI-XRD patterns of the Cu NCs deposited onto the glassy carbon substrate: Cu-Octa, Cu-Cube, Cu–S20, and Cu–S10, from left to right. The intensity ratio {111}/{100} is higher in Cu-Octa compared to Cu-Cube, which is consistent with the main facets exposed being the {111} surfaces.

### Electrocatalytic NO_3_RR Performance

Figure 2 summarizes the electrocatalytic performance of the proposed library of Cu NCs at varying applied potentials. The NO_3_RR chronoamperometry (CA) measurements were performed in 0.1 M NaOH + 10 mM NaNO_3_, which is the most commonly used electrolyte across literatures.^14,15,19,20,26,27^ Cyclic voltammetry (CV) curves of the Cu NCs show the typical redox features of reactants/intermediates, as previously reported (Figure S2).^19^ The product analysis was conducted via UV–vis spectroscopy (Figures S3 and S4).

**Figure 2:**
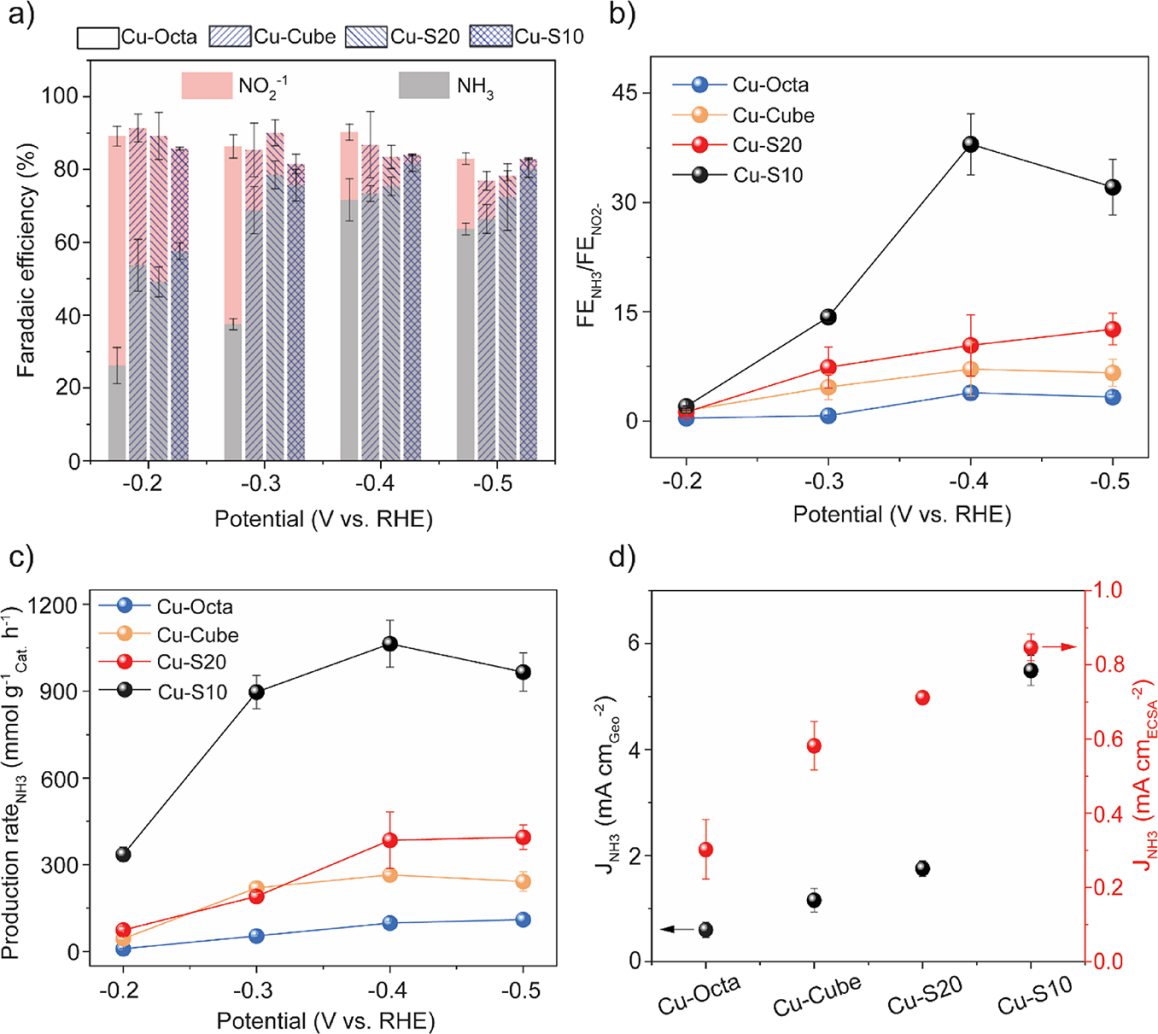
Electrocatalytic performance for NO_3_RR. (a) Faradaic efficiency for NH_3_ and NO_2_^–^; (b) NH_3_ selectivity; and (c) NH_3_ production rate of Cu NCs at studied potentials of −0.2 V_RHE_, −0.3 V_RHE_, −0.4 V_RHE_, and −0.5 V_RHE_ during NO_3_RR; (d) NH_3_ partial current density (left axis) and partial current density normalized by ECSA (right axis) at −0.4 V_RHE_ for Cu-Octa, Cu-Cube, Cu–S20, and Cu–S10. The electrocatalytic NO_3_RR measurements were performed in a H-cell in 0.1 M NaOH + 10 mM NaNO_3_. The error bars are the standard deviations from three freshly prepared electrodes.

Figure 2a shows that all of the studied Cu NCs display a potential-dependent trend in product distribution. As the applied potential shifts more cathodically from −0.2 V_RHE_ to −0.4 V_RHE_, the Faradaic efficiency (FE) for NH_3_ progressively increases and is accompanied by a reduction of the FE for NO_2_^–^. Cu–S10 reaches the highest NH_3_ FE of 80% at −0.4 V_RHE_. No additional increase in FE_NH3_ occurs at −0.5 V_RHE_ due to the enhanced hydrogen evolution at more cathodic potentials, which is consistent with previous studies.^18^

Plotting the ratio of FE_NH3_ to FE_NO2–_ versus the applied potential better highlights the differences among the studied Cu catalysts (Figure 2b). The data clearly indicate that Cu–S10 is the most selective catalyst toward NH_3_ formation over the studied potential region. The production rate of NH_3_ follows the same trend, with the highest NH_3_ production rate exhibited at −0.4 V_RHE_ for the investigated catalyst library (Figure 2c). Notably, the NH_3_ production rate of Cu–S10 is more than 3 times higher than those of the other catalysts in the studied potential region. Specifically, the NH_3_ production rate of Cu–S10 reaches 1064 mmol g_Cat._^–1^ h^–1^at −0.4 V_RHE_, which surpasses those of many recently reported Cu-only catalysts for NO_3_RR (Table S1).

Since the best performances were obtained at −0.4 V_RHE_ for all catalysts, this potential was selected for subsequent evaluation of the catalysts. Figure 2d evidences that Cu–S10 has the highest NH_3_ partial current density (*J*_NH3_) as well as the intrinsic activity (*J*_NH3_ normalized by the electrochemically active surface area, Figures S5–S7), among all the studied catalysts. These results indicate that Cu–S10 possesses specific active sites with increased selectivity and activity for NH_3_. Thus, we proceeded to investigate the features of Cu–S10 that account for such optimal behavior in NO_3_RR.

### Active Catalyst Features Driving NO_3_RR Selectivity toward NH_3_

First, we learned that the surface-bound ligands are stripped by the negative potential and thus do not play any major role in the catalytic performance (Figure S8).

Then, we performed operando X-ray absorption spectroscopy (XAS) measurements to assess the oxidation state of the Cu catalysts during operation as the presence of Cu^+^ or Cu^2+^ under operation has been suggested to impact the activity and selectivity of NO_3_RR.^17,28^ While not providing information specific to the surface, XAS can be performed in the same cell used to evaluate the catalytic performance and, as such, XAS allows for comparison of major compositional changes occurring in the catalyst composition under realistic operational conditions. In the present study, XAS measurements showed that metallic Cu dominates at −0.4 V_RHE_ in all catalysts, despite Cu–S10 being highly oxidized during the start-up phase (Figures S9–S11). Some residual oxides persist in both Cu–S20 and Cu–S10. While surface oxide might contribute to the reactivity of Cu–S10, the data suggest that only differences in the oxidation state of the catalysts under operating conditions are not sufficient to explain the observed differences in selectivity and activity between the samples.

Then, we analyzed the morphology of the Cu NCs after NO_3_RR and compared them to those of the as-synthesized samples with the idea of understanding the impact of the structure/composition of precatalysts on their evolution under operating conditions.

Figure 3 summarizes the results obtained using transmission electron microscopy (TEM) techniques. Specifically, the TEM images evidence that Cu-Octa, Cu-Cube, and Cu–S20 do not undergo major morphological changes (Figure 3a). In contrast, Cu–S10 completely loses its initial morphology and forms a continuous network of particles (Figure 3a). The corresponding electron diffraction (ED) patterns indicate that all catalysts remain crystalline, and the typical reflections of metallic Cu and Cu_2_O are present, the latter due to inevitable oxidation due to air exposure of the samples as well as exposure to water at an open circuit potential after catalysis (Figure 3b). High-resolution TEM (HRTEM) reports the presence of distinct Cu nanograins in Cu–S10 (Figures 3c and S12), with grain boundaries likely enriched in defects.^29–32^ A comparison of the crystallite sizes of Cu NCs before and after the NO_3_RR reveals minimal changes, even for Cu–S10 (Table S2). In agreement with the size analysis, post-electrolysis ECSA analysis shows that all studied Cu NCs retain comparable ECSA values (Figure S13 and Table S3). These results indicate that the average number of active sites in the Cu NCs for the NO_3_RR is mostly preserved even upon reconstruction.

**Figure 3:**
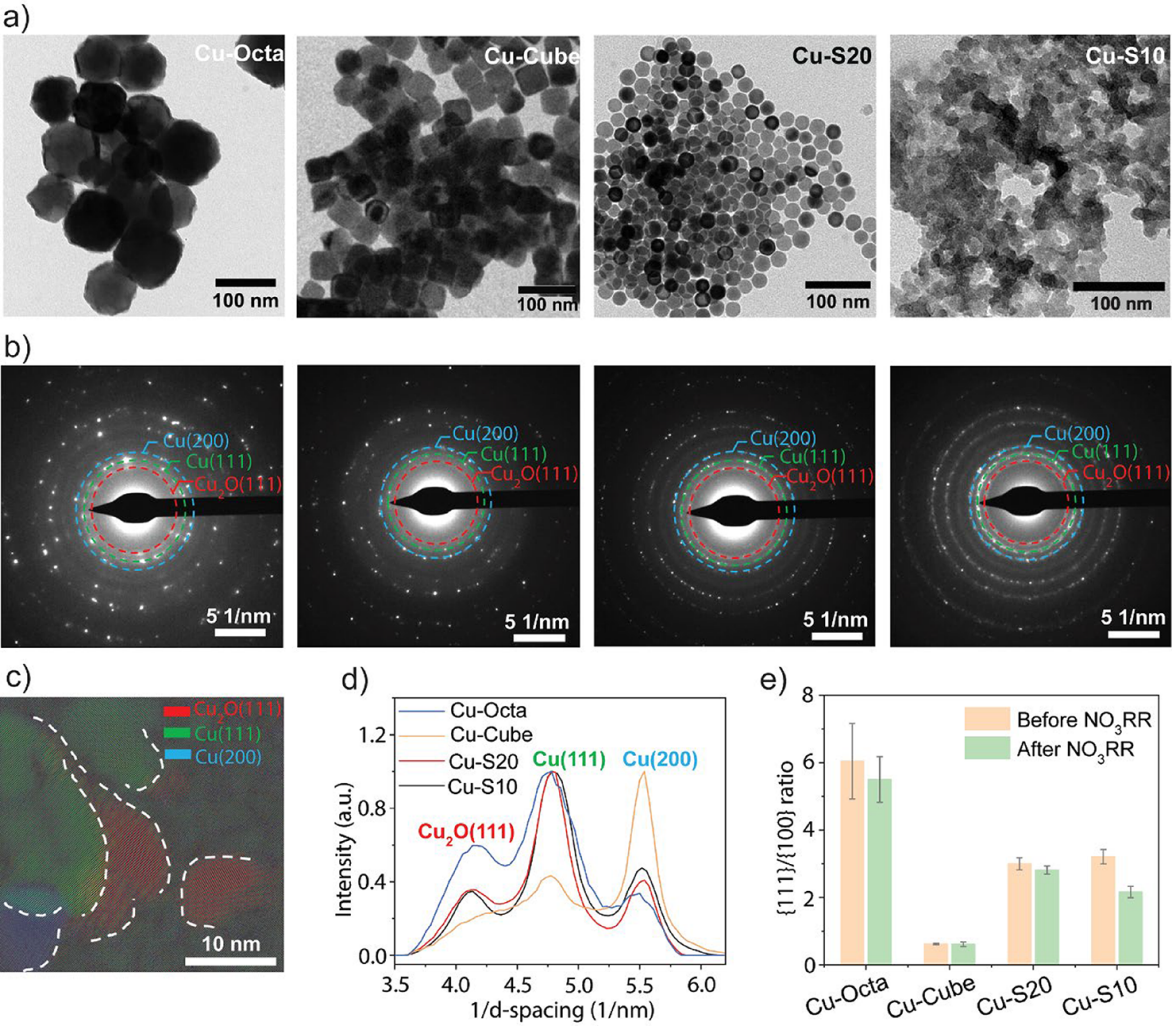
Morphological and structural characterizations of Cu NCs after NO_3_RR. (a) Bright-field TEM images of post-NO_3_RR Cu NCs: Cu-Octa, Cu-Cube, Cu–S20, and Cu–S10 from left to right; (b) corresponding ED patterns of post-NO_3_RR Cu NCs; (c) map of different orientations of Cu–S10 indicates the presence of Cu_2_O­(111) (marked as red), Cu(111) (marked as green), and Cu(200) (marked as blue); (d) ED integrated intensity profiles derived from the ED patterns in (b); (e) comparison of {111}/{100} area ratios before and after NO_3_RR at −0.4 V_RHE_. The reported error bars in (e) are obtained by performing the same analysis in (d) after tilting the same samples to angles of α = 0°, +25°, and −25° relative to the incident electron beam (Figure S15).

Further analysis of the ED patterns provides information regarding the abundance of certain facets in the catalysts. (Figures 3d,e and S14–S16). The Cu (111) and Cu (200) peaks can be visualized by integrating the ED patterns, and the corresponding area can be extracted (Figures 3d and S14). The relative area ratios provide insight into the main exposed facets.^33,34^ The {111}/{100} facet ratios were calculated by dividing the area of the Cu(111) diffraction peak by that of the Cu(200) diffraction peak both for the pristine and the post-electrolysis samples (Figures 3d and S14–S16). The {111}/{100} facet ratios remain relatively constant within the error before and after electrolysis for Cu-Octa, Cu-Cube, and Cu–S20, which indeed exhibit negligible reconstruction during the NO_3_RR. Instead, the {111}/{100} facet ratio shows a decrease from 3 to 2 for Cu–S10, which is consistent with the more severe reconstruction of this sample (Figure 3e). While ED is a bulk technique, surface facet analysis from high-resolution TEM, Pb-UPD, and in-plane grazing incidence diffraction (IPGID) support these results (Figures S12, S17, and S18). Altogether, the data provide a hint that a specific {111}/{100} facet ratio of around 2 in the active catalysts, which results from the structural reconstruction of Cu–S10, may play a role in determining the reactivity of Cu in the NO_3_RR.

To further investigate the importance of a specific facet ratio in the NO_3_RR, we prepared a physical mixture of Cu-Cube and Cu-Octa to achieve a {111}/{100} facet ratio of 2 (Figure S19), which is close to the facet ratio measured for the reconstructed Cu–S10 sample. TEM images show that the Cu-Cube and Cu-Octa are homogeneously mixed with each other, and no major reconstruction occurs after NO_3_RR (Figure 4a), consistently with the behavior of the bare Cu-Cubes and Cu-Octa. The facet ratio was also preserved after the NO_3_RR, as shown by ED analysis (Figure S19). Interestingly, both the selectivity and production rate for NH_3_ of the mixture substantially overcome those of the bare samples with a similar surface area (Figure 4b). This result confirms the importance of the simultaneous presence of the {111} and {100} facets in a certain ratio for the reactivity in NO_3_RR. However, both the NH_3_ selectivity and production rate of the physically mixed sample are approximately only half that of Cu–S10.

**Figure 4:**
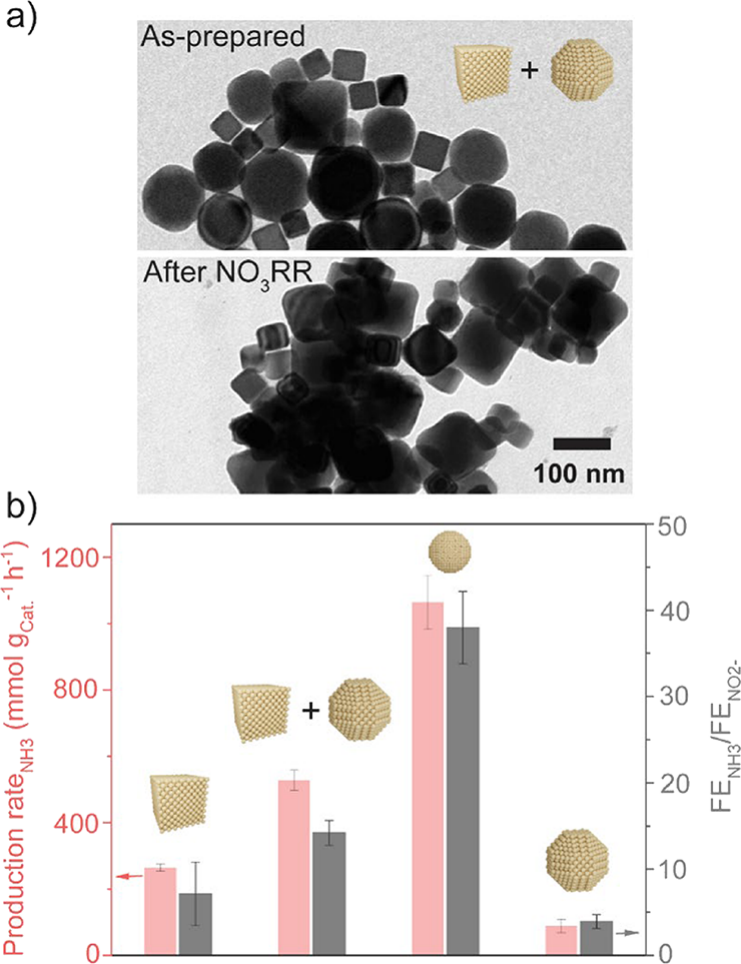
Importance of the {111}/{100} facet ratio. (a) TEM images of the as-prepared and after NO_3_RR physically mixed (Cu-Cube + Cu-Octa) sample; (b) comparison of NO_3_RR performance of the physically mixed sample, the Cu-Octa, the Cu-Cube, and the Cu–S10. The physically mixed sample (Cu-Cube + Cu-Octa) was prepared by mixing Cu-Octa and Cu-Cube with a specific concentration in the catalyst ink to achieve the {111}/{100} facet ratio of 2. NO_3_RR measurements were performed at −0.4 V_RHE_ in the H-cell for 30 min in 0.1 M NaOH + 10 mM NaNO_3_. The error bars are the standard deviations from the three freshly prepared electrodes.

These findings indicate that achieving an optimized facet synergy effect in NO_3_RR requires not only an optimal tuning of the {111}/{100} facet ratio but also the spatial vicinity of the two facets as well as the formation of interfaces (i.e., grain boundaries), which are also important to maximize NH_3_ productivity in NO_3_RR.

### Precatalyst Features Driving Their Reconstruction toward the Best-Performing NO_3_RR Catalysts

Given the unique ability of Cu–S10 to evolve into the most selective and active catalyst for NO_3_RR, we investigated the importance of the precatalyst features behind its structural reconstruction. We considered two factors: (1) the higher relative content of Cu oxide in the smaller spheres and, thus, the more drastic reconstruction induced by its reduction to metallic Cu under cathodic potential; or (2) initial morphological features (e.g., size or shape) which induce higher activity for NO_3_RR and, thus, reconstruction toward the most catalytically active form.

To investigate the impact of Cu oxide on the reconstruction of the precatalysts, we used Cu oxide cubes (Cu_2_O-Cube) as an additional sample (Figure S20). Interestingly, the Cu_2_O-Cube sample undergoes reconstruction into a network, which resembles Cu–S10 (Figure 5a). The operando XAS data are also similar for the two samples (Figures S21 and S22). Concomitantly, the NH_3_ production rate of Cu_2_O-Cube is much higher compared to that of the metallic Cu-Cube, while still not reaching that of the Cu–S10 (Figure 5c). In order to deconvolute the impact of nitrate and cathodic potential on the precatalyst reconstruction, we conducted a pretreatment consisting of performing LSV in the absence of nitrate for Cu_2_O-Cube and Cu–S10. TEM images indicate that structural reconstruction is similar to that observed under nitrate-containing conditions occurs (Figure 5a,b). These reconstructed samples exhibit a similar NH_3_ production rate to that of the samples reconstructed in the presence of nitrate (Figure 5c). This result highlights the importance of the cathodic potential, rather than the NO_3_RR itself, in driving the reconstruction of the precatalysts.

**Figure 5:**
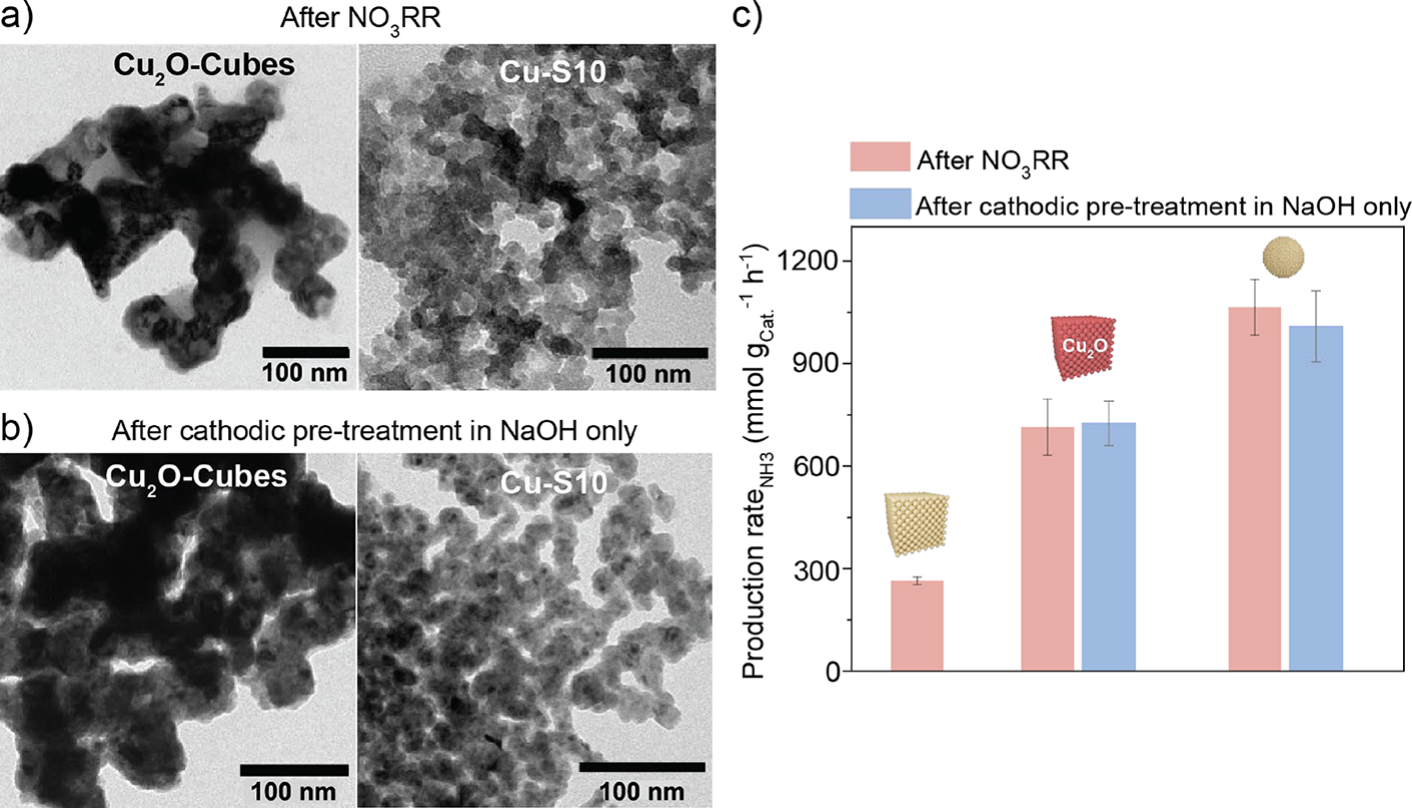
Importance of the oxide fraction. (a,b) Bright-field TEM images of the Cu_2_O-Cubes and Cu–S10 after pretreatment with applied cathodic potential via LSV in NaOH + NaNO_3_ and in NaOH, respectively. (c) NH_3_ production rates for the Cu-Cube, Cu_2_O-Cubes, and Cu–S10 pretreated with LSV in NaOH + NaNO_3_ (red histograms) and for the Cu_2_O-Cube and Cu–S10 pretreated with LSV in NaOH (blue histograms). The NO_3_RR electrolysis data were acquired in 0.1 M NaOH + 10 mM NaNO_3_ at −0.4 V_RHE_ for 30 min.

### Mechanistic Insight

Altogether, the results above evidence that the copresence of {111} and {100} facets (i.e., NC shape) and their spatial vicinity (i.e., NC size) along with the initial presence of oxide are the key features of the Cu precatalysts to maximize the NH_3_ production rate in the NO_3_RR (Figure 6a).

**Figure 6:**
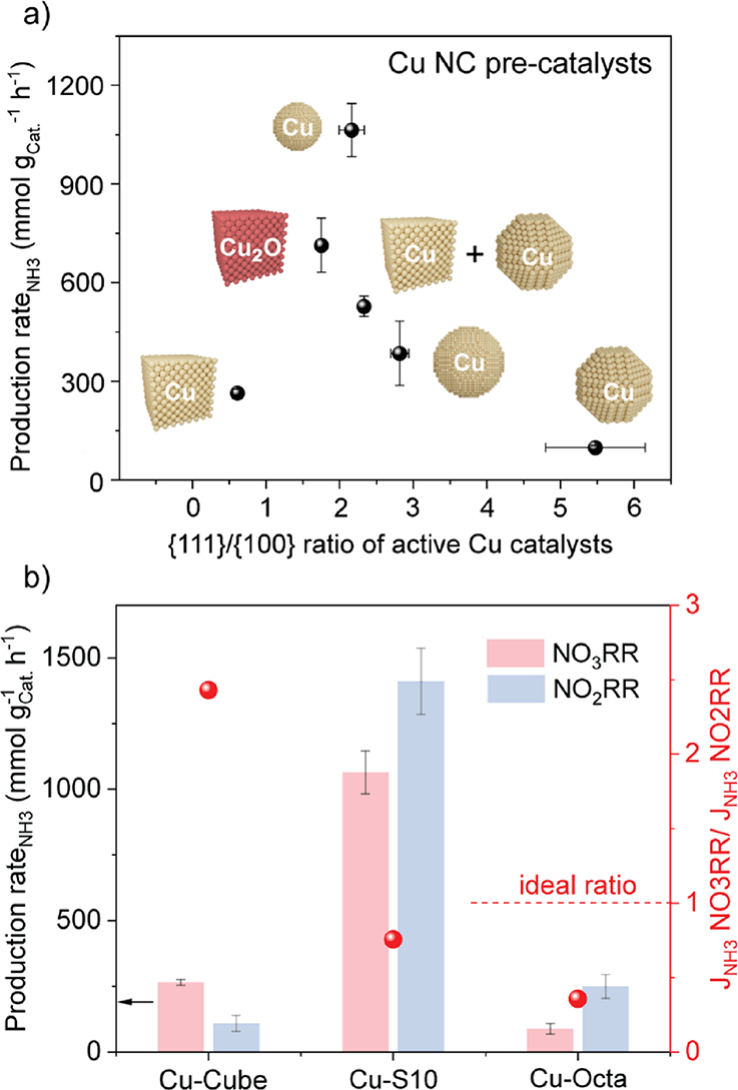
Maximizing ammonia production in NO_3_RR via an optimal {111}/{100} facet ratio and spatial proximity of these facets. (a) Schematic of the NH_3_ production rate versus the {111}/{100} facet ratio in the active Cu catalysts for all the colloidal Cu NCs used as precatalysts. (b) Comparison of NO_3_RR and NO_2_RR performance for Cu-Cube, Cu-Octa, and Cu–S10 precatalysts which evolve into Cu active catalysts with {111}/{100} facet ratios of c.a*.* 0.6, 2.0, and 5.5, respectively. These data indicate that an optimal facet ratio and spatial proximity are obtained when using 10 nm Cu nanospheres as precatalysts and corroborate a tandem mechanism wherein a balanced conversion rate of NO_3_^–^ on the {100} facets and of the NO_2_^–^ intermediate on the {111} facets explains the boost in the NH_3_ production rate.

The coexistence of {100} and {111} facets has been previously suggested to play a synergistic role in NO_3_RR, wherein {100} primarily facilitates the reduction of NO_3_^–^ to NO_2_^–^, while {111} is more effective in catalyzing the subsequent hydrogenation of NO_2_^–^ to produce NH_3_.^18^ This work contributes to advancing the current knowledge in catalyst design for NO_3_RR by suggesting that efficient NO_2_^–^ intermediate utilization occurs only when {100} and {111} facets are in an optimal ratio and within an optimal spatial distance from one other (Figure S23). This optimal ratio and distance can be achieved by the size, shape, and composition of the Cu NCs acting as precatalysts.

We corroborated this literature-based hypothesis by performing NO_2_RR (Figure 6b). An ideally equal conversion rate of NO_3_^–^ and of the NO_2_^–^ intermediate (i.e., *J*_NO3RR_/*J*_NO2RR_ = 1) should maximize the NH_3_ production rate in NO_3_RR within the proposed facet-based tandem scheme. The results reveal that the NH_3_ production rate when NO_3_RR is replaced with NO_2_RR decreases with Cu-Cube and increases with Cu-Octa, which is consistent with the {100} facets facilitating NO_3_^–^ to NO_2_^–^ and the {111} facets being more effective in catalyzing the subsequent hydrogenation of NO_2_^–^ to produce NH_3_. The intrinsic preference for one or the other reaction of these two faces results in an unbalanced *J*_NO3RR_/*J*_NO2RR_. In comparison, Cu–S10 exhibits the highest NH_3_ production rate for both NO_2_RR and NO_3_RR and, importantly, a *J*_NO3RR_/*J*_NO2RR_ closest to the ideal ratio of 1.

This observation highlights the critical importance of a balanced {100}/{111} facet ratio and spatial proximity for an effective tandem mechanism in the NO_3_RR to maximize the NH_3_ production rate.

## Conclusions

In conclusion, this study employed a library of well-defined Cu to elucidate the compositional and structural characteristics of the precatalysts impacting ammonia production in NO_3_RR. The shape, the size, and the oxidation state of the Cu precatalysts emerge as key features driving their reconstruction toward the active catalyst. The main lesson learned is that an optimal {111}/{100} facet ratio and spatial distance of these facets in the active copper catalyst are required to maximize selectivity and activity toward ammonia. Colloidally synthesized 10 nm Cu spheres integrate these key features, achieving a competitive NH_3_ production rate compared to the state-of-the-art.

The obtained fundamental insight deepens the current understanding of the performance-driving parameters for NO_3_RR catalysts along with proposing an approach to tune and achieve the in situ formation of active catalysts with high NH_3_ selectivity and production rate via controlling the size, shape, and composition of colloidally synthesized NC precatalysts.

This work proposes a conceptual framework that can be envisioned beyond NO_3_RR and applied to other catalytic transformations.

## Supplementary Material



## Data Availability

Experimental raw data are openly available in Zenodo at 10.5281/zenodo.17122752.

## Notes

Additional experimental data; chemicals used, Cu NCs synthesis methods, electrochemical measurement protocols, liquid product quantification methods; size statistics, LSVs, CVs, CAs and ECSA determination of Cu NCs; XPS spectra; XANES fitting; XANES analysis of Cu NCs; NO_2_RR measurements; additional TEM images, ED patterns and ED integrated intensity profiles of Cu NCs; and IPGID patterns of Cu NCs after NO_3_RR (PDF)
