# Supplementary material for: Well-Defined Cu Precatalysts Indicate Design Rules for Reactivity in Nitrate Electroreduction
Source: J Am Chem Soc. 2025 Sep 22;147(39):35438–45. doi: 10.1021/jacs.5c09246 (PMC12498389; doi:10.1021/jacs.5c09246)
Supplement: Supplementary file 1 [file ja5c09246_si_001.pdf]

Supporting Information

Well-defined Cu pre-catalysts indicate design rules for reactivity in nitrate electroreduction

Jia Du[a], Anna Loiudice[a], Krishna Kumar[a], Ludovic Zaza[a], Raffaella Buonsanti[a,*]

a. Laboratory of Nanochemistry for Energy (LNCE), Institute of Chemical Sciences and Engineering (ISIC), École Polytechnique Fédérale de Lausanne, CH-1950 Sion, Switzerland.

* Corresponding author: raffaella.buonsanti@epfl.ch

Chemicals and Materials

All chemicals were used as received. copper(I) bromide (CuBr, 99.999%), copper(I) acetate (Cu(OAc), 98%), oleylamine (OLAM, 70%), tri-noctylamine (TOA, 98%), Tetradecylphosphonic acid (TDPA, 98%), Tri-n-octylphosphine oxide (TOPO, 99%), tri-n-octylphosphine (TOP, 90%), Bis(2,4,6-trimethylphenyl)phosphine, phenol nitroprusside solution, alkaline hypochlorite solution, standard NH_4Cl solution (analytical standard), standard $NaNO_2$ solution (99%), sodium hydroxide (NaOH ≥98.0%), sodium nitrate ($NaNO_3$, ≥99.0%), nitrite test kit, maleic acid (≥99.0%), toluene (anhydrous, 99.8%) and hexane (anhydrous, 95%) were purchased from Sigma-Aldrich. The proton exchange membrane (Frontcell^{TM} PFSA) was purchased from Frontis Energy.

Synthesis of Cu NCs

Synthesis of Cu-Cube: The synthesis of Cu-Cube followed a reported procedure.[1] Briefly, TOPO (940 mg, 2.4 mmol) was added in a 25 mL flask and kept under vacuum for 30 min at room temperature. OLAM (70%, 11.6 mL, 24.6 mmol) was added to the mixture and heated it to 120°C for 30 min under vacuum. Afterwards, the solution was cooled down to room temperature under N_2 atmosphere. CuBr (99.999%, 15.2 µL, 0.5 mmol) was then added and the temperature was quickly increased to 260°C. The solution was heated at 260°C during1 h under N_2 atmosphere before being cooled down to room temperature naturally. The solution was then transferred via glass vials under N_2 to a glovebox and divided into two centrifuge tubes. Approximately 6 mL EtOH was added to each tube, and then centrifuged at 13000 rpm for 10 min. The precipitate in each tube was collected in 4 ml hexane, diluted with 4 mL EtOH and centrifuged once again at 13000 rpm for 10 min. Finally, the precipitate was stored in 2 mL toluene in a glovebox.

Synthesis of Cu-S10: Cu-S10 was synthesized following a method adapted from previous procedures.[2] Briefly, TOA (12.5 ml) was added in a 25 mL flask and kept under vacuum for 30 min at room temperature. Cu(OAc) (153 mg, 1.25 mmol) and TDPA (174 mg, 0.625 mmol) were added and the mixture was heated to 180°C for 30 min under N_2. Afterwards, the solution was further heated to 270°C for 30 min under N_2 before being cooled down to room temperature naturally. The solution was then transferred via glass vials under N_2 to a glovebox followed and divided into two centrifuge tubes. Approximately 6 mL EtOH was added to each tube, and then centrifuged at 13000 rpm for 10 min. The precipitate in each tube was collected

in 4 mL hexane, diluted with 4 mL EtOH and centrifuged once again at 13000 rpm for 10 min. Finally, the precipitate was stored in 2 mL toluene in a glovebox.

Synthesis of Cu-S20: In a glovebox, CuBr (99.999%, 65 mg, 0.45 mmol), bis(2,4,6-trimethylphenyl)phosphine (120 mg, 0.45 mmol) and pre-degassed OLAM (70%, 14 mL, 42.4 mmol) were added to a 50 mL 3-neck flask. The flask was sealed and quickly connected to a Schlenk line under N_2. The mixture was heated to 60°C for 5 min under vacuum. Then, the temperature was quickly increased to 285°C under N_2 with a heating ramp of 25-30°C/min. The solution was heated at 285°C during 5 min under N_2 atmosphere before being cooled down to room temperature. The solution was then transferred via a glass vial under N_2 to a glovebox and put into a centrifuge tube. Approximately 7.5 mL of hexane and 7.5 mL EtOH were added to the tube, and then centrifuged at 13000 rpm for 10 min. The precipitate was collected in 5 mL hexane, diluted with 5 mL EtOH and centrifuged once again at 13000 rpm for 10 min. Finally, the precipitate was stored in 2 mL toluene in a glovebox.

Synthesis of Cu-Octa: Cu-Octa was synthesized following a procedure modified from the literature.[3,4] In a glovebox, CuBr (98%, 115 mg, 0.8 mmol), TOP (450 µL, 1.0 mmol) and pre-degassed OLAM (80-90%, 15.0 mL, 45.4 mmol) were added to a 50 mL 3-neck flask. The flask was sealed and quickly connected to a Schlenk line under N_2. The mixture was heated to 110°C for 30 min under vacuum. The solution was then kept an extra 30 min at 110°C under N_2. The temperature was quickly increased to 270°C with a heating ramp of 25-30°C/min. The solution was heated at 270°C during 1h under N_2 atmosphere before being cooled down to room temperature. The solution was then transferred via a glass vial under N_2 to a glovebox and put into a centrifuge tube. Approximately 15 mL toluene was added to the tube, and then centrifuged at 13000 rpm for 10 min. The precipitate was collected in 10 mL toluene and centrifuged once again at 13000 rpm for 10 min. Finally, the precipitate was stored in 2 mL toluene in a glovebox.

Synthesis of Cu_2O-Cube: Cu_2O-Cube was synthesized following a method reported previously.[5] In brief, 0.5 mL of $CuSO_4 \cdot 5H_2O$ solution (0.1 M) was added to 45.75 mL of Milli-Q water in a 100 mL beaker and the mixture was stirred for 10 min. Afterwards, 1.75 mL of 1 M NaOH solution was poured to the mixture under vigorous stirring, this step was followed by the addition of the freshly prepared L-ascobic acid solution (0.25 M) of 2 mL. Finally, the mixture was further stirred at toom temperature for 15 min. The reaction solution was washed and

centrifuged 3 times at 13000 rpm for 10 min, each time with 5 mL of ethanol and 5 mL of Milli-Q water. At the end, the final product was collected in 5 mL of ethanol for further analysis.

Catalyst characterizations

Transmission electron microscopy (TEM): Bright-field TEM images and corresponding electron diffraction (ED) patterns were acquired on a FEI Tecnai-Spirit operated at 120 kV. High resolution TEM (HRTEM) images were acquired on Thermo Fisher Scientific Spectra 200 operated at 200 kV. Prior to imaging, the as-synthesized Cu NCs were diluted with hexane and drop-casted on a copper TEM grid (Ted Pella, Inc.), and the Cu NCs after electrochemistry was transferred to the copper TEM grid by smearing the TEM grid on glassy carbon on which the Cu NCs were deposited. At least five areas were independently selected for each sample analysis. ED patterns were acquired by configuring TEM to diffraction mode, this followed by the insertion of a selected area diffraction (SAD) aperture into the TEM column. The orientation of Cu particles depends on how they are deposited onto the TEM grid that may generate an error in the measurements. To take this effect into account, we acquired TEM images and corresponding electron diffraction at three different angles of $\alpha = 0°, +25°, -25°$ relative to the incident electron beam. The obtained integrated patterns generate three independent measurements and therefore an error of the measurement could be extracted. ED patterns were analyzed using the Crystallographic Tool Box software (CrysTBox-Server 1.10).[6] ED integrated intensity profiles were derived from these patterns using the same software. Subsequent analysis involved deconvolution, fitting, and integration of diffraction planes on circular averaged radial diffraction profiles that correspond to Cu(111), Cu(200), and Cu_2O(111), respectively, to determine the integrated area of each diffraction plane. The facet ratios {111}/{100} discussed in the study were extracted from above analysis.

Grazing incidence X-ray diffraction (GIXRD): The Cu NCs films onto glassy carbon before NO_3RR were investigated through GIXRD. GIXRD measurements were performed using a Bruker D8 Discover Plus instrument equipped with a Cu rotating anode and a Dectris Eiger2 detector. The experimental setup included a primary 2.5° axial Soller slit and a secondary 0.5° equatorial Soller slit positioned along the beam path. All samples were measured at the incidence angle of 0.1°, with a 0.2 mm divergence slit employed to condition the X-ray beam.

In plane grazing incidence diffraction (IPGID): The Cu NCs films onto glassy carbon after NO_3RR were investigated through IPGID. IPGID measurements were performed using a

Bruker D8 Discover Plus instrument equipped with a Cu rotating anode and a Dectris Eiger2 detector. The beam was shaped with a focussing Göbel mirror (60 mm), followed by a 0.1 mm selection slit and an axial 0.5° Soller slit on the primary side. 2Theta scans were performed using the 0D detector mode with an active sensor area of 140 x 200 px. The beam was accepted using an axial 1.0° Soller slit (equatorial, if considering scan direction). Data were acquired at 0.1° incidence angle, 5s/step and a step size of 0.08°.

Operando X-ray absorption spectroscopy (XAS): XAS experiments were conducted at Swiss-Norwegian beamlines (BM31) of the European Synchrotron Radiation Facility (ESRF) in Grenoble, France. The catalyst suspension was deposited onto a glassy carbon substrate (2.5 × 2.5 × 0.5 mm) via drop-casting to reach the catalyst loading of 40 μg_{Cu} cm^{-2}, and a Kapton window was used to facilitate X-ray transmission. Cu standards were analysed in transmission mode and in situ XAS measurements were carried out in fluorescence mode. The Cu K-edge X-ray absorption near edge structure (XANES) spectra were obtained by averaging all spectra collected over a 30 min time interval. The measurements started with linear sweep voltammetry conducted between 0.7 V and -0.7 V vs RHE in 0.1 M NaOH + 10 mM $NaNO_3$ at a scan rate of 20 mV s^{-1}. After, chronoamperometry measurements were performed at -0.2 V_{RHE}, -0.3 V_{RHE} and -0.4 V_{RHE} for 30 min.

Inductively coupled plasma optical emission spectroscopy (ICP-OES): The concentration of Cu NPs solution was quantified using ICP-OES performed with an Agilent 5100 instrument. For each sample, a 10 μL aliquot of the solution was initially dried and subsequently digested overnight in 285 μL of 70% HNO_3. The digested sample was then diluted with Milli-Q water to a final volume of 10 mL. Calibration standards with Cu concentrations of 0.1, 0.5, 1, 5, and 10 ppm were freshly prepared from a 1000 ppm Cu standard reference solution in 2 wt.% HNO_3 prior to each analysis session.

X-ray photoelectron spectroscopy (XPS): The measurements were carried out using an Axis Supra instrument (Kratos Analytical) equipped with a monochromated Al Kα X-ray source. Spectra were acquired with a pass energy of 40 eV and a step size of 0.15 eV. Samples were electrically insulated from the sample holder, and charge compensation was applied during analysis. All binding energies were calibrated to the C 1s peak of the C-C bond at 284.8 eV.

Electrochemical measurements

All electrochemical measurements in present study were performed using a computer controlled potentiostat (Biologic SP-300). An H-type cell made of polyether ether ketone (PEEK), which comprises cathodic and anodic compartments, was used for measurements. The working electrode (WE) was prepared by drop-casting Cu nanoclusters (NCs) ink onto the centre of a glassy carbon (GC), which exposed a circular area of 1.33 cm^2 for measurements. The loading of Cu NCs onto GC was 20 μg_{Cu} cm^{-2}. A platinum foil and an Ag/AgCl electrode (leak free series, Innovative Instruments, Inc.) were used as the counter electrode (CE) and reference electrode (RE), respectively. The voltage between the Ag/AgCl RE and a commercial reversible hydrogen electrode (RHE, HydroFlex®) was measured for 10 min in 0.1 M NaOH aqueous solution and the last value measured was used to convert the measured potential to the RHE scale. The cathodic part of the H-cell was filled with 0.1 M NaOH + 10 mM $NaNO_3$ solution serving as electrolyte, while the anodic compartment was filled with 0.1 M NaOH solution, for each compartment the volume of electrolyte was 2.5 mL. A pre-activated proton exchange membrane was utilized to separate the two compartments.

The NO_3RR measurements were performed under ambient conditions at room temperature. Argon gas was continuously bubbled into electrolyte (cathodic compartment) during measurements to get rid of oxygen. The measurement protocol for current study includes the following steps: a. Linear sweep voltammetry (LSV) measurements, b. Electrochemical impedance spectroscopy (EIS) measurements, c. Determination of electrochemical double layer (ECDL), d. Chronoamperometry (CA) measurements, e. Determination of ECDL once again.

Initially, LSV were performed between 0.7 V and -0.7 V vs RHE in 0.1 M NaOH + 10 mM $NaNO_3$ at a scan rate of 20 mV s^{-1} to remove residual ligands on the surface of Cu NCs. Note that an extended potential of -1 V vs RHE was needed to remove ligands on Cu-S20 surface. Subsequently, the used electrolyte was replaced with fresh electrolyte in the cathodic compartment. This was followed by EIS measurements to determine the cell resistance between WE and RE, conducted over a frequency range of 1 MHz to 100 Hz with an amplitude of 20 mV. Next, the ECDL of the electrode was measured to determine the ECSA of Cu NCs. Briefly, CVs were recorded at increasing scan rates between 4 and 32 mV s^{-1} within the potential window of 0.78 to 0.82 V vs Ag/AgCl, where no faradaic processes occur. The difference in current density between cathodic and anodic sweeps at 0.8 V vs Ag/AgCl scales linearly with

scan rate, and the slope of this linear relationship was used to calculate the double layer capacitance (C_{DL}). The ECSA was then calculated by dividing C_{DL} by the capacitance of GC (C_{GC} = 23.4 μF/cm²).[7] For the chronoamperometry experiments, a LSV was performed from 0.25 V vs RHE to the target potential at a scan rate of 5 mV s^{-1}, this was followed by holding potential at the target value for 0.5 h. The spent electrolyte from both compartments of the H-cell was collected for product quantification.

The NO_2RR measurements were performed under the same conditions of the NO_3RR measurements, with the only difference being the substitution of NO_3^- with an equivalent concentration (10 mM) of NO_2^-. LSVs were performed between 0.7 V and -0.7 V vs RHE in 0.1 M NaOH + 10 mM $NaNO_2$ at a scan rate of 20 mV s^{-1} to remove residual ligands on the surface of Cu NCs. After, the electrolyte was changed and chronoamperometry measurements were performed at -0.4 V vs RHE for 30 min. The electrolyte from both anode and cathode compartment of the H-cell was collected for product quantification.

Pb UPD experiments were conducted in the H-cell in the Ar-saturated solution of 0.1 M NaOH + 3 mM $Pb(ClO_4)_2$. The scan rate was 20 mV s^{-1}. 5 CV cycles were performed and the 3rd CV was analysed for comparison

Liquid product quantification

Determination of ammonia: Spectrophotometric indophenol-blue method was used to quantify NH_3 produced during NO_3RR. Briefly, 60 μL of electrolyte taken from cathodic part was diluted with Milli-Q water to 2 mL, then, 500 μL of alkaline hypochlorite solution and 500 μL of phenol nitroprusside solution were added to the diluted electrolyte. The mixture was incubated in the dark for 30 min before recording the UV-Vis spectra. Similarly, the potential presence of NH_3 in anodic part was analyzed as well, allowing the total amount of NH_3 produced during NO_3RR to be determined as the sum from both compartments. The calculation curve was established using standard NH_4Cl solution with different concentrations (Figure S2). The faradaic efficiency of NH_3 (FE_{NH3}) and the production rate of NH_3 (Production $rate_{NH3}$) were calculated following equation 1 and equation 2, respectively.

$$FE_{NH3} = \frac{(8F \times V \times c_{NH3})}{(Q \times MM_{NH3})} \times 100 \qquad \text{(equation 1)}$$

$$Production\ rate_{NH3} = \frac{(V \times c_{NH3})}{(m_{Cat.} \times t \times MM_{NH3})} \qquad \text{(equation 2)}$$

Where F is the Faradaic constant (96485 C/mol), c_{NH3} is the mass concentration of the aqueous NH_3 (mg/L), V is the volume of electrolyte in individual compartment of H-cell (2.5 mL), Q is the charge passed during CA measurement, MM_{NH3} is the molar mass of NH_3, $m_{Cat.}$ is the total catalyst loading within the 1.33 cm^2 circle and t is the holding time of NO_3RR electrolysis.

Determination of nitrite (NO_2^-): Spectrophotometric method was used to quantify NO_2^- produced during NO_3RR. Briefly, 40 μL of electrolyte taken from cathodic part was added to a glass vial containing 35 mg of white powder from a commercial nitrite test kit, subsequently, 40 μL of H_2SO_4 (diluted 100 times from concentrated 98% H_2SO_4) was added to the aforementioned mixture, this procedure was followed by adding Milli-Q water to reach 4 mL. The mixture was then incubated for 20 min in the dark before UV-Vis analysis. The calculation curve was established using standard $NaNO_2$ solution with different concentrations (Figure S3). The faradaic efficiency of NO_2^- (FE_{NO2-}) calculated following equation 3.

$$FE_{NO2-} = \frac{(2F \times V \times c_{NO2-})}{(Q \times MM_{NO2-})} \times 100 \qquad \text{(equation 3)}$$

Where F is the Faradaic constant (96485 C/mol), c_{NO2-} is the mass concentration of NO_2^- (mg/L), V is the volume of electrolyte in the cathodic compartment (2.5 mL), Q is the charge passed during CA measurement, MM_{NO2-} is the molar mass of NO_2^-.

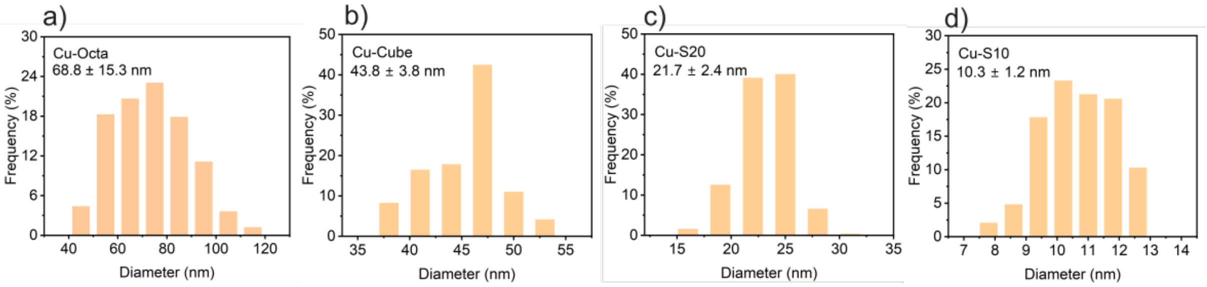

Figure S1. (a-d) Cu particle size distribution determined from TEM images by counting at least 200 individual particles of Cu-Octa (a), Cu-Cube (b), Cu-S20 (c) and Cu-S10 (d).

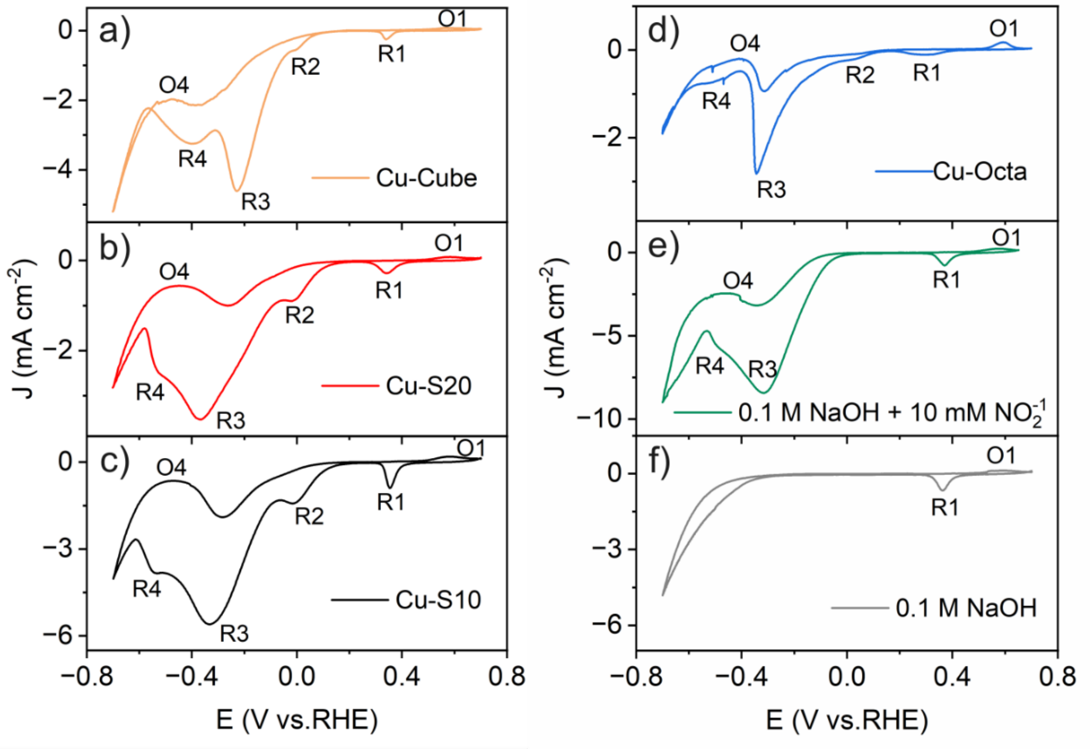

Figure S2. (a-d) CVs of the studied Cu NCs recorded in 0.1 M NaOH + 10 mM NO_3^- and (e, f) CVs of Cu-S10 recorded in 0.1 M NaOH + 10 mM NO_2^- and in 0.1 M NaOH. The measurements were performed in an H-type cell with a scan rate of 20 mV s^{-1}.

The quasi-reversible R1/O1 redox peaks, corresponding to the Cu(I)/Cu(0) transition, are observed in the CVs of all studied catalysts, irrespective of the presence or absence of NO_3^-. The cathodic peak area of R1 serves as an indicator for the electrochemical surface area of Cu catalysts, with Cu-S10 exhibiting the largest R1 peak area, consistent with the trend shown in Fig. S7 and Table S3. Two irreversible cathodic peaks, R2 and R3, are attributed to the stepwise electroreduction of NO_3^-.[5] Specifically, R2 is assigned to the reduction of NO_3^- to NO_2^-, supported by its disappearance when NO_3^- is replaced with NO_2^- in the electrolyte. R3 is ascribed to the further reduction of NO_2^- to nitrogen-containing products with lower oxidation states.[5] The quasi-reversible R4/O4 peaks likely arise from the adsorption/desorption of *OH species on the catalyst surface, in agreement with previous study.[5]

We observed that the shape and position of the cathodic peaks are dependent on the catalyst used. This behaviour is likely attributed to differences in shape and size, which influence ECSA and surface roughness, thereby affecting the double-layer capacitance. Variations in capacitive contributions can broaden the peaks and shift their apparent positions. However, no prior studies have systematically reported the influence of catalyst shape and size on the

characteristics of the peaks in CVs under NO_3RR conditions for our reference, thus conclusions are premature at this time.

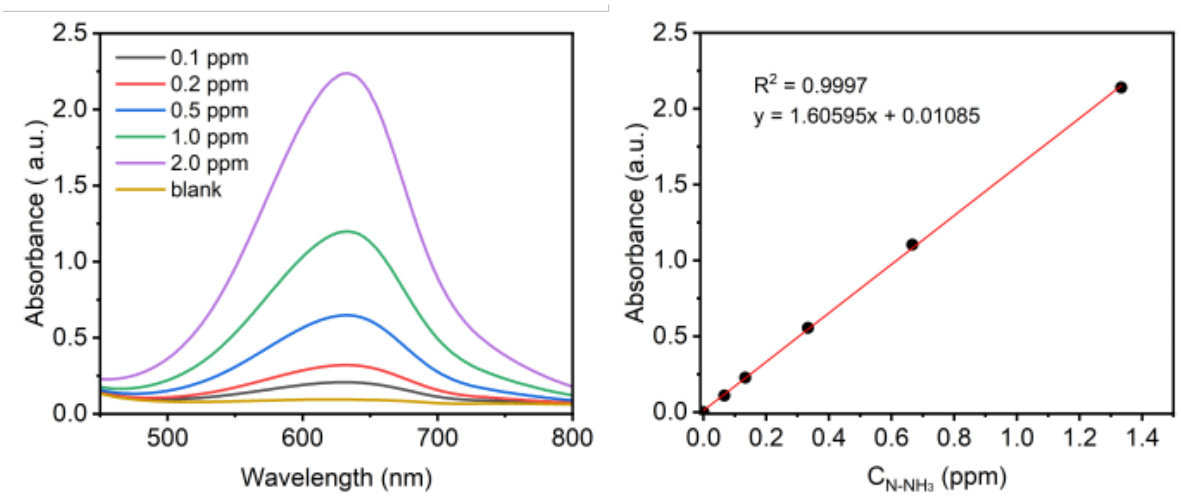

Figure S3. Calibration curve for NH_3 quantification using spectrophotometric blue indophenol method.

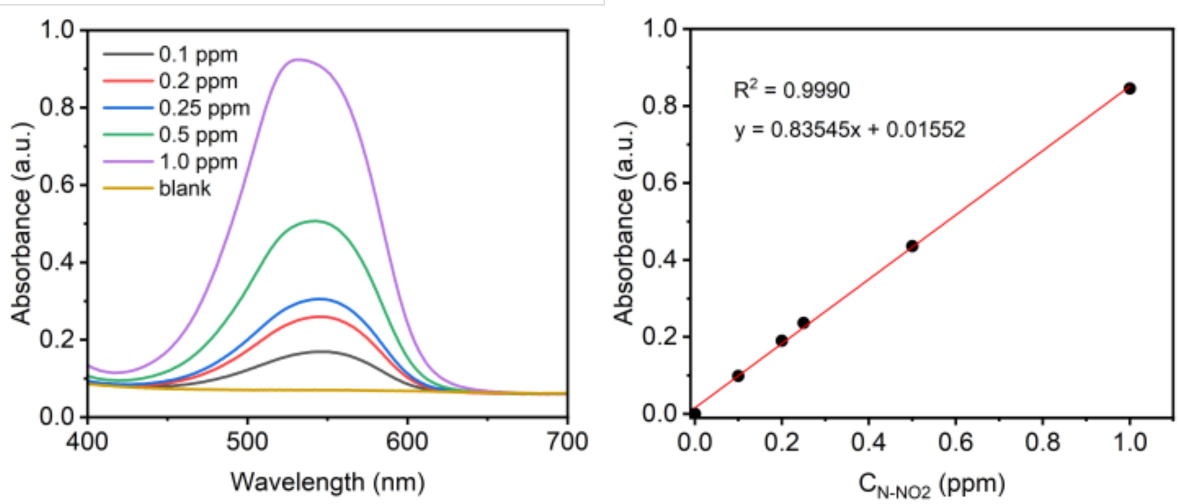

Figure S4. Calibration curve for NO_2^- quantification using spectrophotometric method.

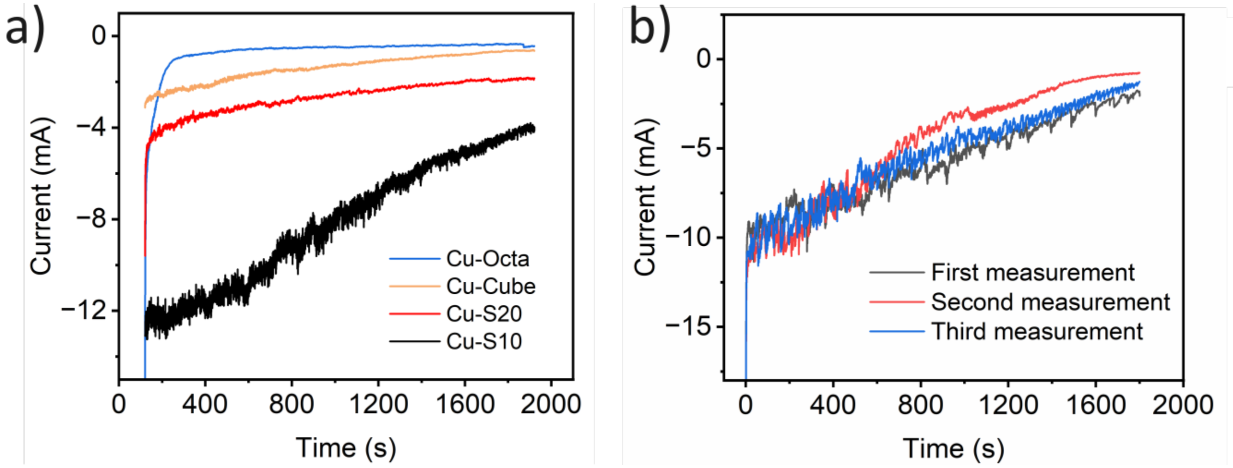

Figure S5. (a) Current versus time at constant applied potential of -0.4 V_{RHE} for all studied Cu NCs; (b) three cycles of CA for Cu-S10 performed at -0.4 V_{RHE}, each cycle was performed upon rinsing the electrode, the electrochemical cell and replacing the electrolyte.

We note the occurrence of a gradual decay of current, more evident in the case of Cu-S10 (Figure 5a). This decay can be attributed to the depletion of NO_3^-, being more pronounced for the most active Cu-S10. In addition, previous studies suggest that current decay may also arise from the accumulation of strongly adsorbed reaction intermediates on catalyst surface, such as $*NO_2$ and *NO, which can block active sites and hinder further reaction.[8] To verify these hypotheses, we performed three additional CA measurements upon replacing the electrolyte with fresh one and rinsing the electrode and the electrochemical cell at each cycle (Figure S5b). The obtained data show that the current at time zero is recovered during the three cycles suggesting that the decrease in current is not due to catalyst degradation rather to the above mentioned reasons.

Table S1. Recently reported NH_3 production rate from NO_3RR on single Cu catalysts.

Catalysts	Electrolyte	Potential	NH_3 yield	NH_3 faradaic efficiency	Ref.
Cu nanosheets	0.1 M KOH + 10 mM KNO_3	-0.15 V_{RHE}	22.95 mmol g_{cat}^{-1} h^{-1}	99.7%	9
Cu Nanosheets	1 M KOH + 0.2 M KNO_3	-0.59 V_{RHE}	621.8 mmol g_{Cu}^{-1} h^{-1}	~88%	10
Cu oxide nanobelts (100)	1 M KOH + 0.1M NO_3^{-1}	-0.15 V_{RHE}	650 mmol g_{cat}^{-1} h^{-1}	95%	11
Cu_2O nanocubes	0.1 M Na_2SO_4 + 8 mM $NaNO_3$ (pH 12)	-0.3 V_{RHE}	480 mmol g_{cat}^{-1} h^{-1}	~88%	5
CuO_x nanoparticles	0.1 M KOH + 50 ppm KNO_3	-0.25 V_{RHE}	26.45 mmol g_{cat}^{-1} h^{-1}	~74.2%	12
Cu(111) nanodisks	0.1 M KOH + 10 mM KNO_3	-0.5 V_{RHE}	82.4 mmol g_{cat}^{-1} h^{-1}	81.1%	13
Cu single-atom doped BCN	0.1 M KOH + 100 mM NO_3^{-1}	-0.4 V_{RHE}	1539.7 mmol g_{Cu}^{-1} h^{-1}	97.28%	14
Cu–N_4 single-atom catalyst	0.1 M KOH + 0.1 M KNO_3	-1.0 V_{RHE}	12500 mmol g_{Cu}^{-1} h^{-1}	84.7%	15
10 nm Cu spheres	0.1 M NaOH + 10 mM $NaNO_3$	-0.4 V_{RHE}	1064 mmol g_{Cat}^{-1} h^{-1}	82%	This work

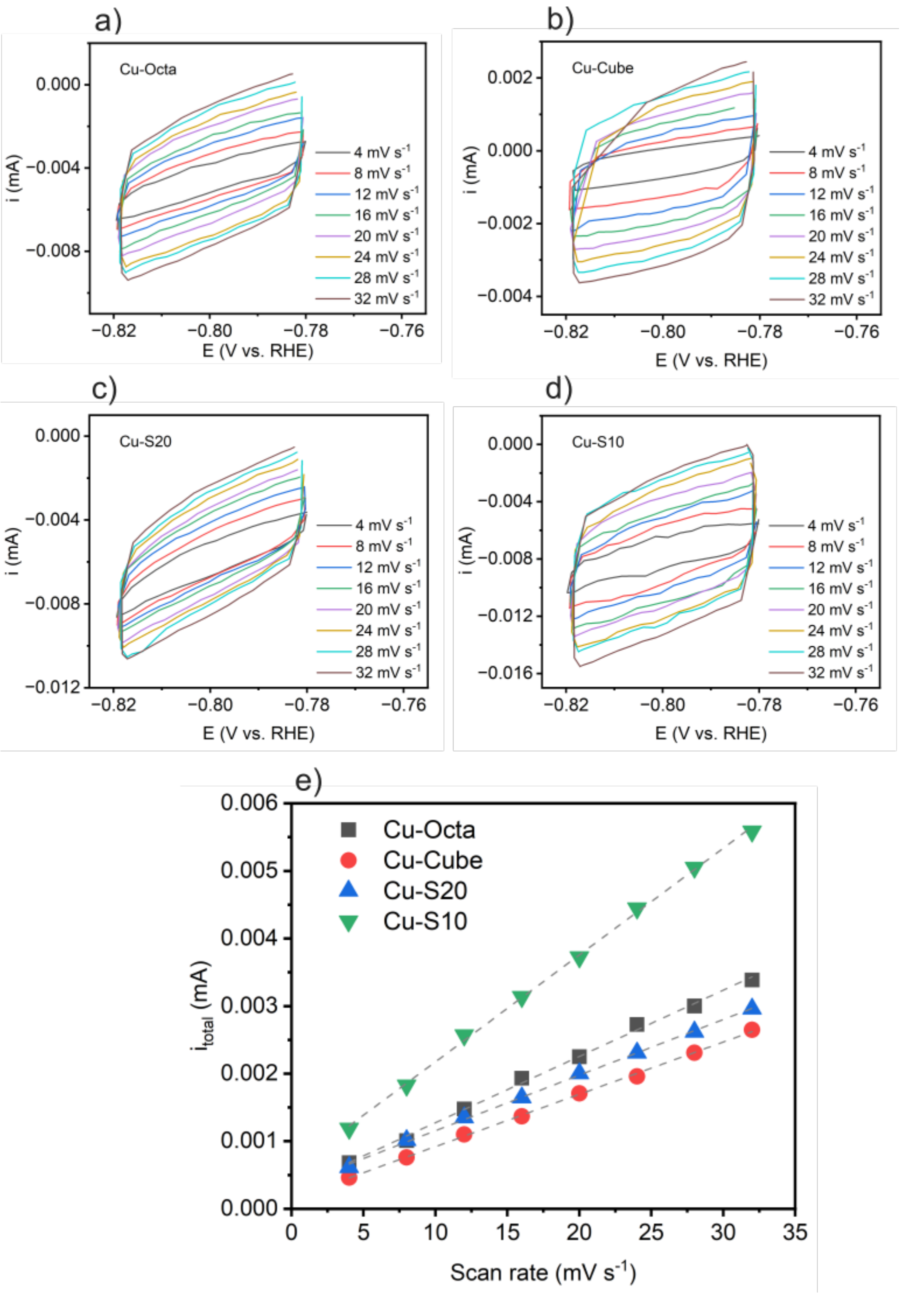

Figure S6. (a-d) Representative cyclic voltammograms used to determine capacitance and ECSAs before NO_3RR measurements for Cu NCs of Cu-Octa (a), Cu-Cube (b), Cu-S20 (c), Cu-S10 (d) and (e) i_{total} plotted against scan rate to determine the capacitance of Cu NCs.

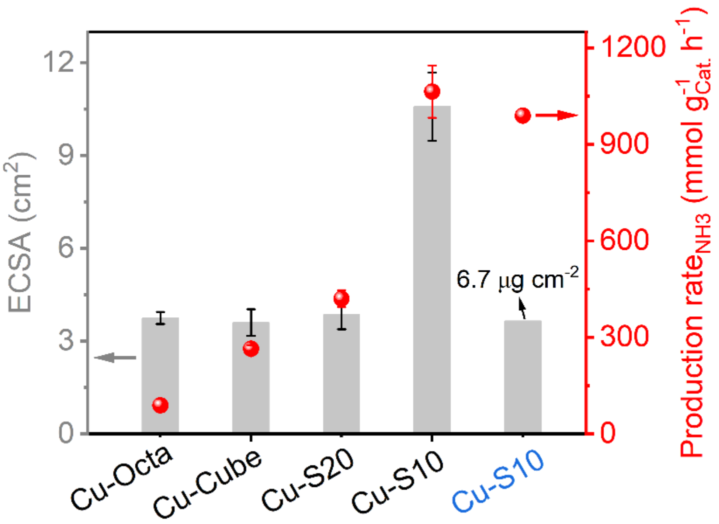

Figure S7. Comparison of ECSA and NH_3 production rate at -0.4 V_{RHE} for catalysts with a loading of 20 μg cm^{-2} onto glassy carbon (Cu-Octa, Cu-Cube, Cu-S20, and Cu-S10) and 6.7 μg cm^{-2} onto glassy carbon (Cu-S10, marked in blue). All electrochemical measurements were conducted in an H-cell with the cathodic compartment containing 0.1 M NaOH + 10 mM $NaNO_3$. ECSAs were collected before NO_3RR chronoamperometry experiments. Error bars represent the standard deviation based on three independently prepared samples.

Variations in the size (i.e. surface-to-volume ratio) of Cu NCs can impact the catalytic activity as they will result in different ECSA (i.e. higher ECSA for smaller NCs). A higher ECSA provides more active sites for reactant/intermediates adsorption, which can enhance the measured electrocatalytic activity.[16] Therefore, we evaluated the ECSAs of Cu NCs by analysing their double-layer capacitance via cyclic voltammetry at different scanning rates (Figure S6).[17] The resulting values show that Cu-S10 exhibits a higher ECSA compared to the other samples at the same catalyst loading (Figure S7). However, the fact that the NH_3 reduction rate remains unchanged even when the ECSA of Cu-S10 is reduced by decreasing the catalyst loading to match that of the other samples. This suggests that the enhanced catalytic performance of Cu-S10 is independent of particle size effect.

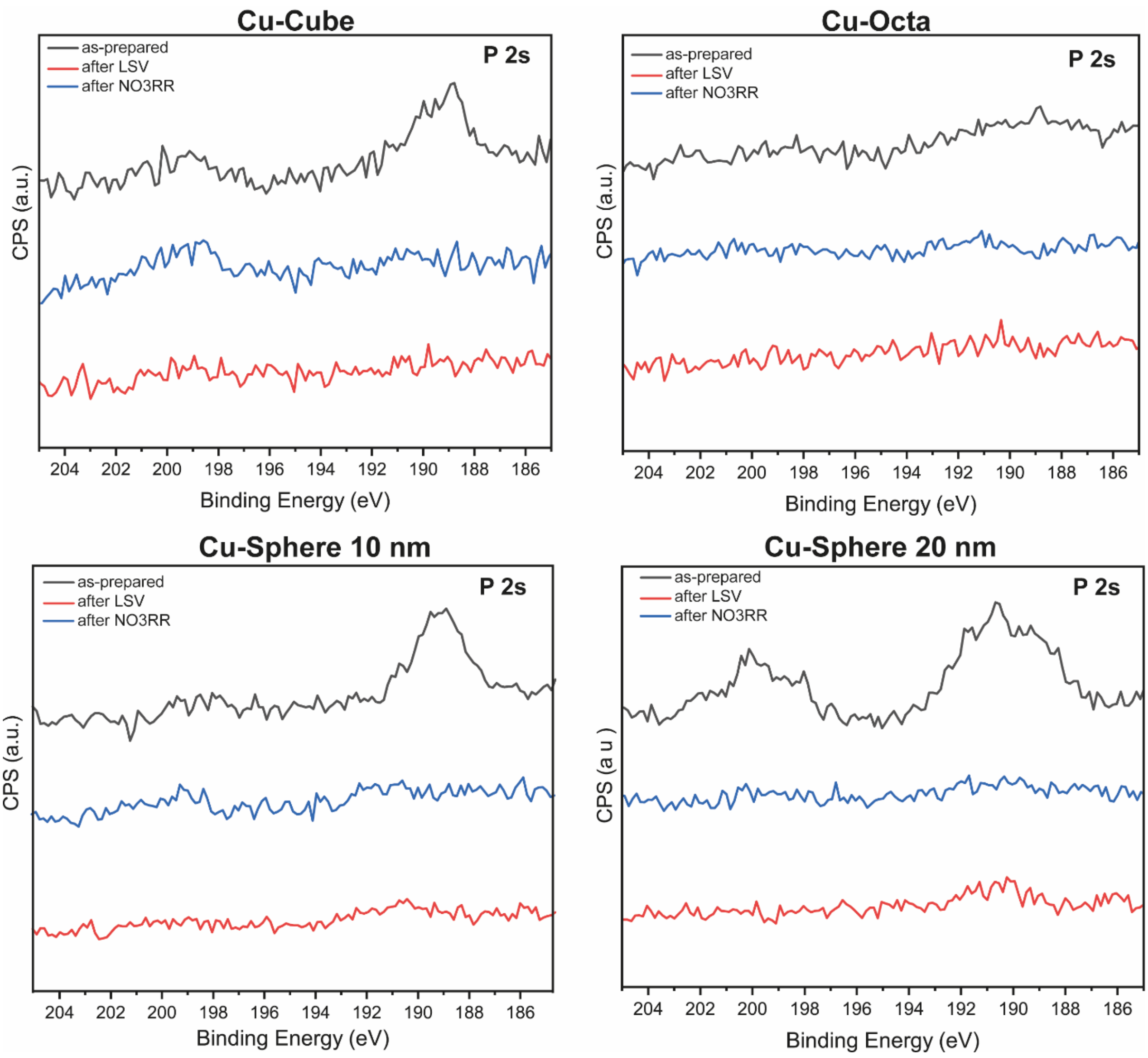

Figure S8. XPS spectra of the P 2s core level of the studied Cu NCs in three stages: as-prepared, after LSV treatment and after NO_3RR at -0.4 V_{RHE} for 30 min.

To evaluate the presence of ligands on Cu catalyst surface and, thus, to assess any ligand-related effects on catalytic performance, we conducted XPS measurements targeting the P 2s core level, as all ligands used in the catalyst synthesis are phosphorus-based. Figure S8 shows that the P 2s signals become negligible for all catalysts already during the application of the cathodic potential in the LSV. Consequently, all catalysts operate with "clean surfaces" during the following chronoamperometry measurements and expose a fully inorganic surface for nitrate reduction.

Operando XANES Analysis

Figures S9-S11, S21-S22 summarize the results of the operando XANES analysis. The goal of this analysis was to assess the composition (i.e. fraction of Cu and Cu oxide) of the sample during NO_3RR at -0.4V_{RHE}, which is the potential we chose to compare the performance in this study. Thus, these data suggest that metallic Cu is the dominant phase at -0.4 V_{RHE} under NO_3RR conditions for all samples, while not excluding the contribution of surface copper oxides to the observed behaviour of Cu-S10.

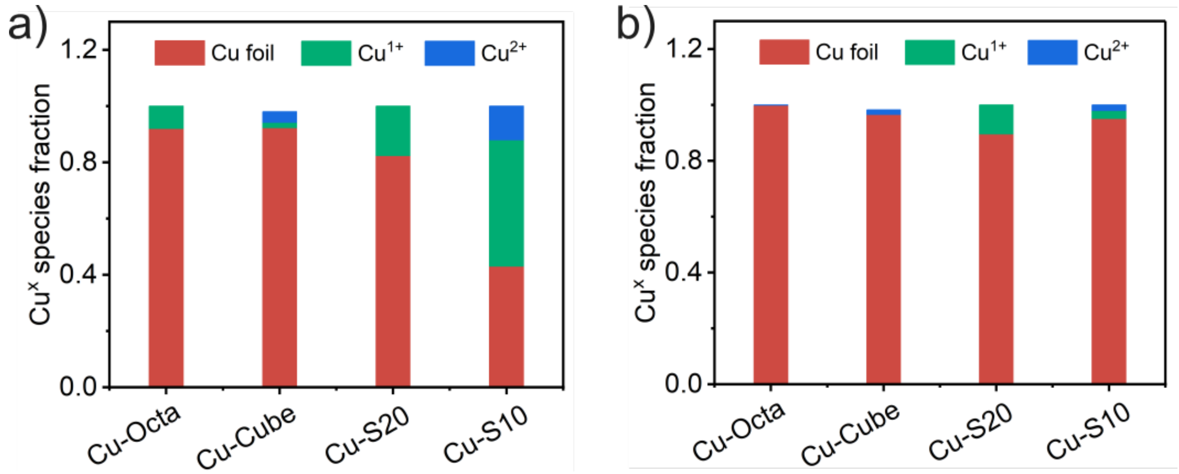

Figure S9. (a,b) Linear combination analysis (LCA) for the tested Cu NCs acquired in operando at OCP (a) and at -0.4 V_{RHE} (b).

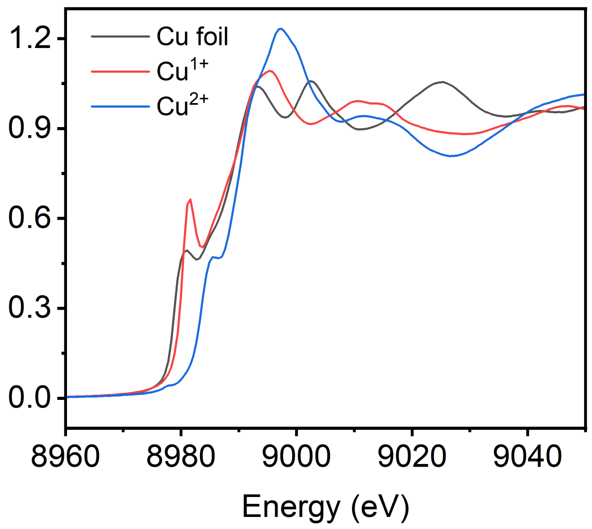

Figure S10. Cu K-edge XANES spectra of Cu, Cu_2O and CuO standards used to build the LCA models in Figure S11.

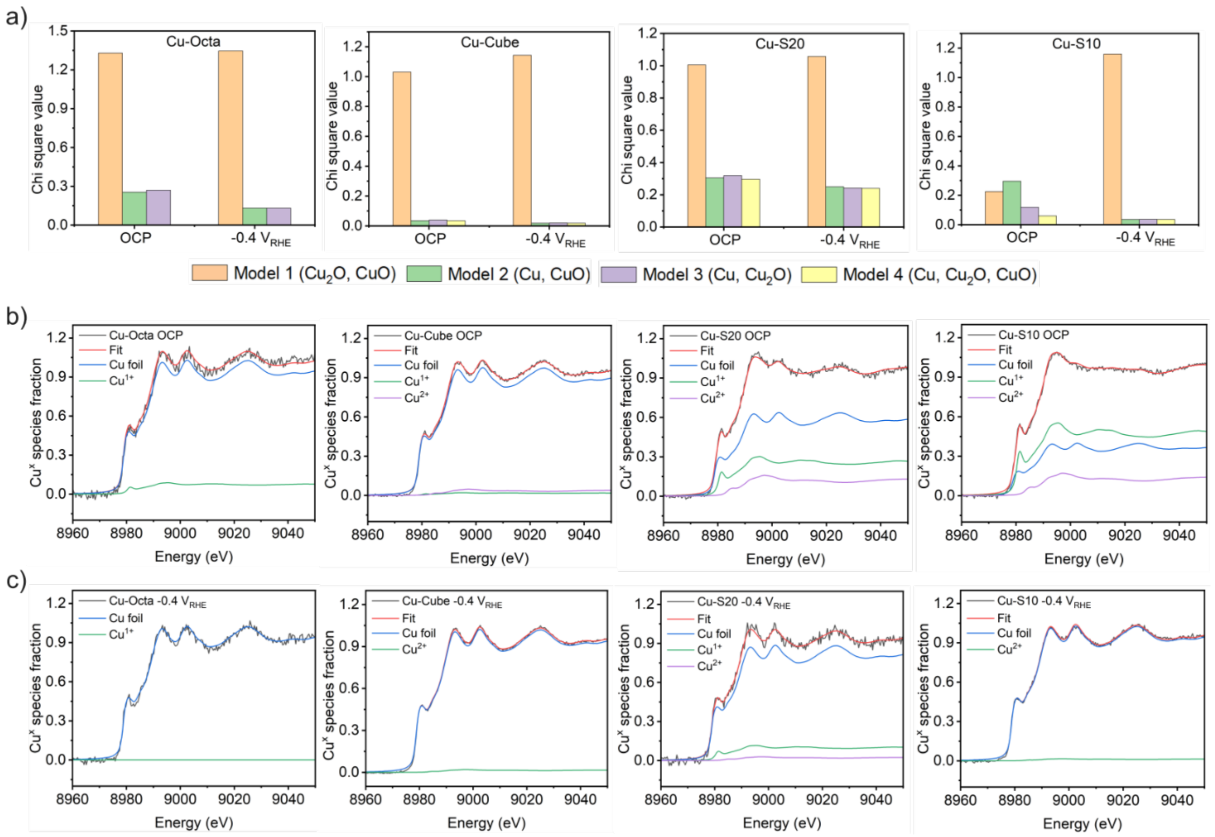

Figure S11. (a) Chi square values at OCP and at -0.4 V_{RHE} for the studied Cu NCs and the four LCA models evaluated based on the standards reported in Figure S10; (b, c) XANES spectra together with the fitted spectra using Model 3 for Cu-Octa and Model 4 for Cu-Cube, Cu-S20 and Cu-S10 measured at OCP (b) and at -0.4 V_{RHE} (c)

The XANES data were normalized using the Larch package. The four LCA models were built by considering different components of copper oxides. At OCP, Cu-Cube, Cu-S20 and Cu-S10 are best modelled using Model 4, as their corresponding chi square values are smallest compared to other Models, while the chi square values by using Model 2 and Model 3 are comparable for Cu-Octa. At -0.4 V_{RHE}, Model 1 that excludes the presence of metallic copper, is not applicable for any systems, due to its highest chi square. This indicates that the metallic copper component must be included at -0.4 V_{RHE}. Models 2, 3 and 4 can all be applied for the studied catalysts at -0.4 V_{RHE}, with Cu-Octa being best modelled using Model 3, and Cu-Cube, Cu-S20 and Cu-S10 being best modelled using Model 4. Therefore, the corresponding best Models are picked for the studied catalysts measured at OCP and -0.4 V_{RHE} for the analysis in Figure S9.

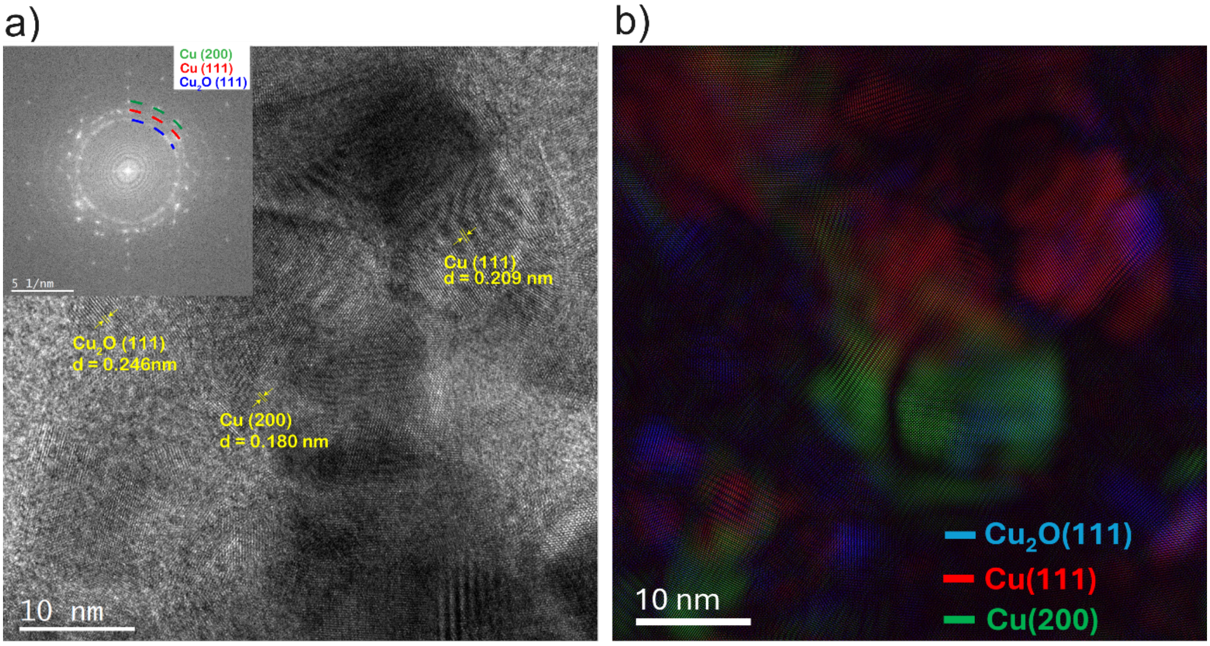

Figure S12. (a) HRTEM image of Cu-S10 after NO_3RR at -0.4 V_{RHE} with lattice spacing extracted from FFT and (b) corresponding map of different orientations.

Table S2. Crystallite size of Cu NCs extracted from grazing incidence X-ray diffraction. Crystallite sizes from Rietveld refinement were based on metallic Cu phase.

Catalysts	Crystallite size (nm)	
	Before NO_3RR	After NO_3RR
Cu-Octa	-	-
Cu-Cube	37	34
Cu-S20	21	16
Cu-S10	9	7

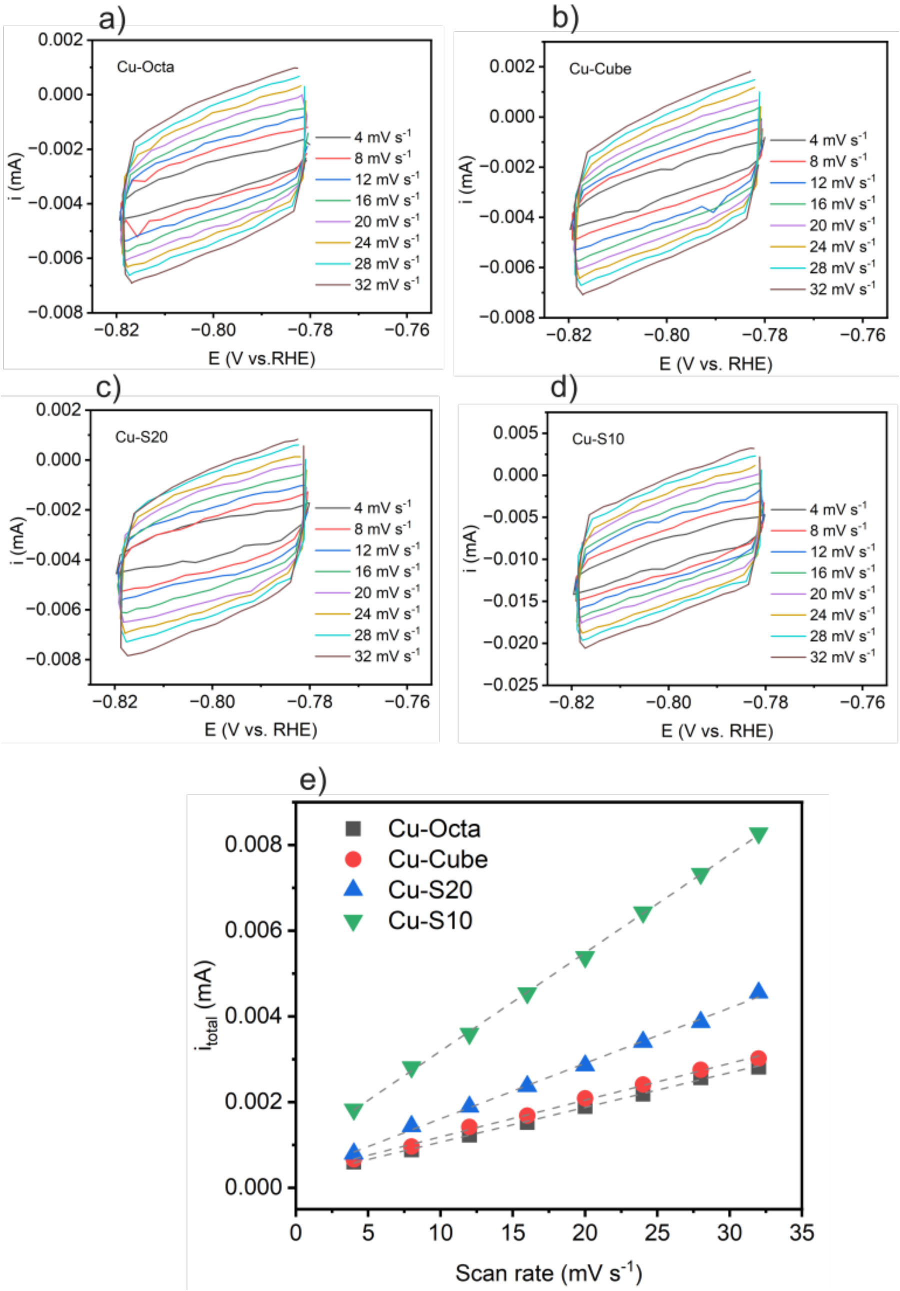

Figure S13. (a-d) Representative cyclic voltammograms used to determine capacitance and ECSAs after NO_3RR measurements for Cu NCs of Cu-Octa (a), Cu-Cube (b), Cu-S20 (c), Cu-S10 (d) and (e) i_{total} plotted against scan rate to determine the capacitance of Cu NCs.

Table S3. Comparison of ECSA of Cu NCs before and after NO_3RR.

Cu NCs	ECSA (cm^2)	
	Before NO_3RR	After NO_3RR
Cu-Octa	3.75 ± 0.19	3.54 ± 0.24
Cu-Cube	3.60 ± 0.43	3.22 ± 0.26
Cu-S20	4.60 ± 0.14	4.10 ± 0.28
Cu-S10	10.58 ± 1.10	9.96 ± 0.56

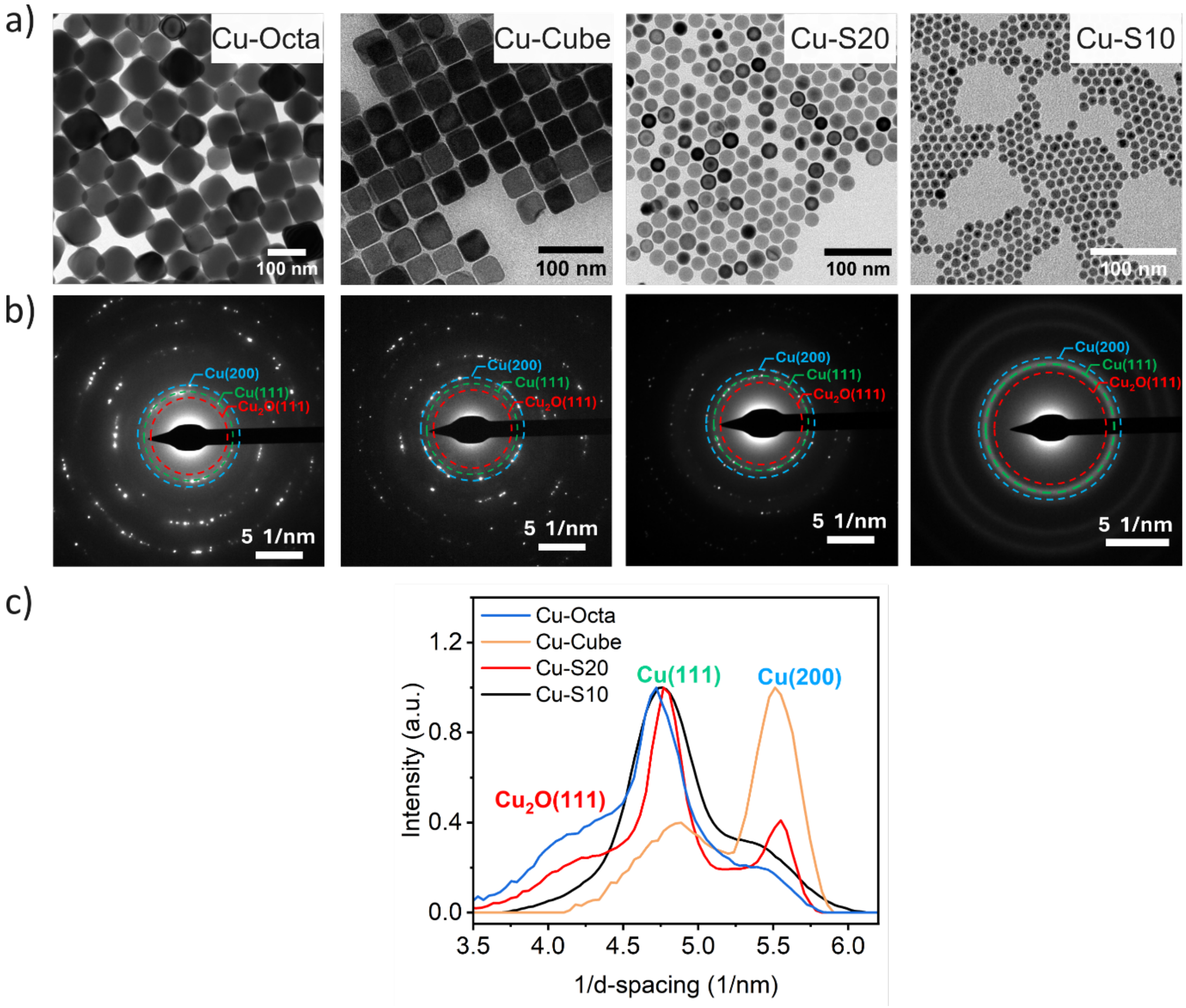

Figure S14. (a) Bright-field TEM images of as-prepared Cu NCs: Cu-Octa, Cu-Cube, Cu-S20 and Cu-S10 from left to right and (b) corresponding ED patterns. (c) ED integrated intensity profiles derived from the ED patterns shown in (b). The presence of Cu_2O in the samples can be caused by unavoidable exposure to air.

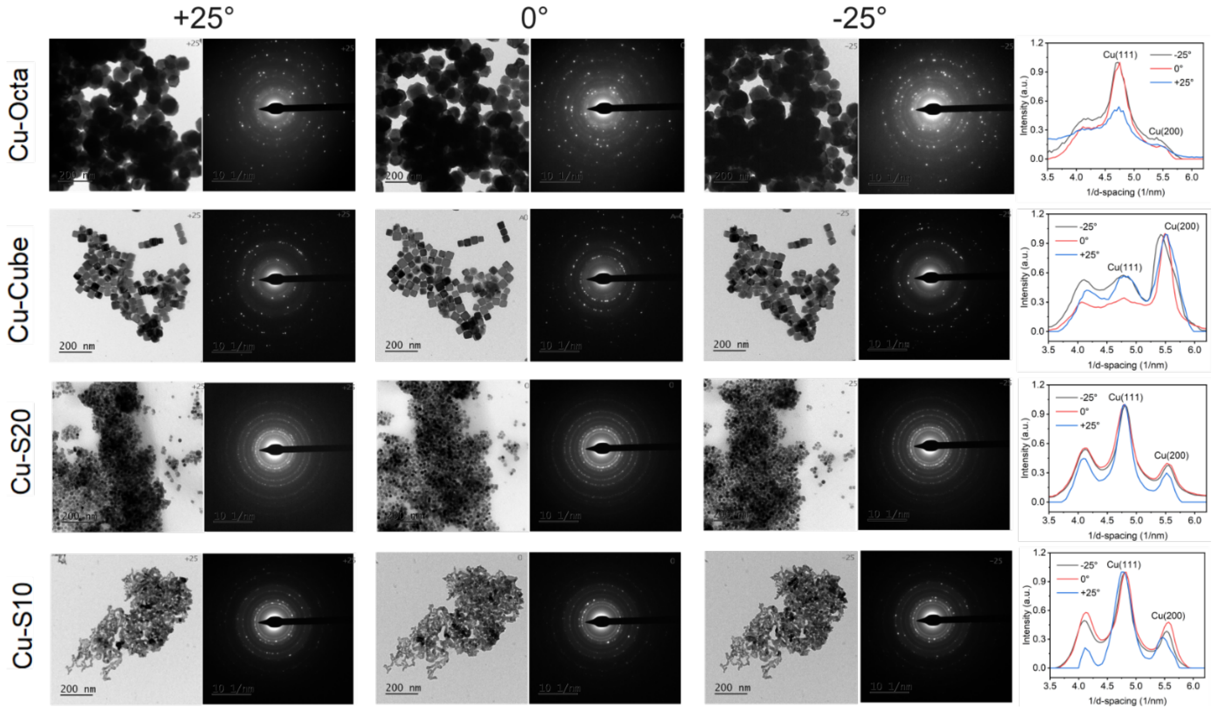

Figure S15. Bright-field TEM images, corresponding ED patterns and ED integrated intensity profiles of Cu catalysts after NO_3RR measurements at -0.4 V_{RHE}. The samples were tilted to different angles with $\alpha = 0°$, +25° and -25°, relative to the incident electron beam.

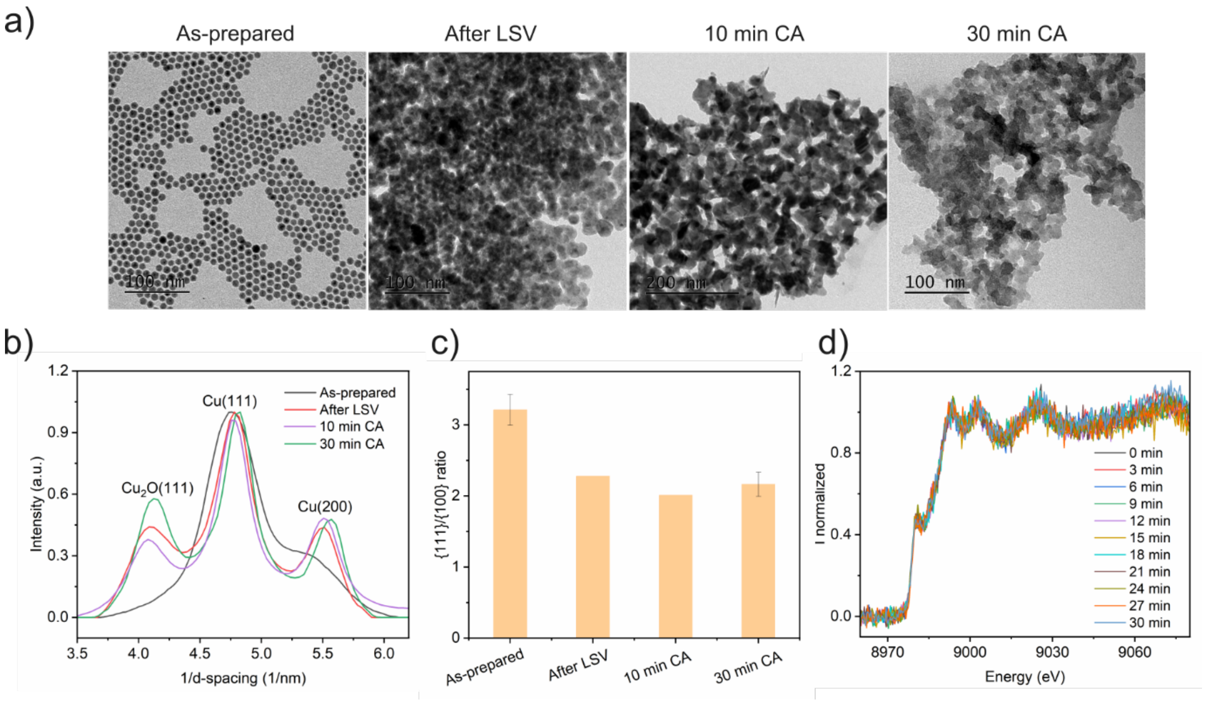

Figure S16. (a) TEM images of Cu-S10: as-prepared, after LSV, after 10 min and 30 min of CA for NO_3RR, (b) corresponding ED integrated intensity profiles, (c) {111}/{100} facet ratios

extracted from ED and (d) evolution of XANES spectra as function of time at -0.4 V_{RHE} for Cu-S10 in 0.1 M NaOH + 10 mM $NaNO_3$ in a H-cell.

Figure S16 illustrates that Cu-S10 undergoes a significant morphological and compositional transformation after the initial LSV scan. The morphology in the form of a continuous network, the facet ratio and the composition (i.e. metallic copper) remain stable during CA for NO_3RR.

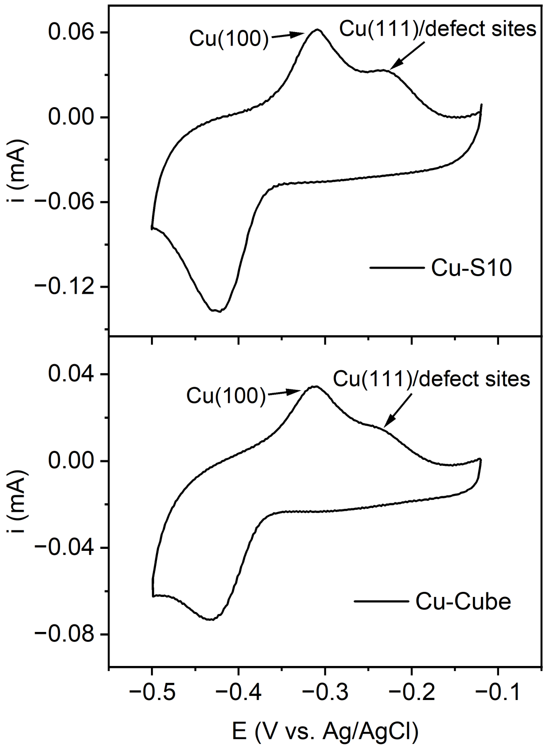

Figure S17. Pb UPD experiments for Cu-S10 and Cu-Cube after 30 min NO_3RR at -0.4 V_{RHE}. These experiments were conducted in a Ar-saturated solution of 0.1 M NaOH + 3 mM $Pb(ClO_4)_2$. The scan rate was 20 mV s^{-1}. 5 CV scans were performed and the 3rd CV was analysed for comparison among the two samples.

Cu-S10 after NO_3RR, thus already in its activated state, exhibits a more pronounced peak associated with Cu(111) compared to Cu-Cube, which is consistent with a higher relative {111}/{100} ratio. However, we note that the Cu(111) peak is convoluted with signals arising from defect sites.[18,19] This overlap renders an accurate quantification of the surface facets via Pb-UPD challenging. Indeed, previous studies employ Pb-UPD only as a qualitative tool to compare the structural characteristics of Cu-based catalysts during/after NO_3RR.[10,20]

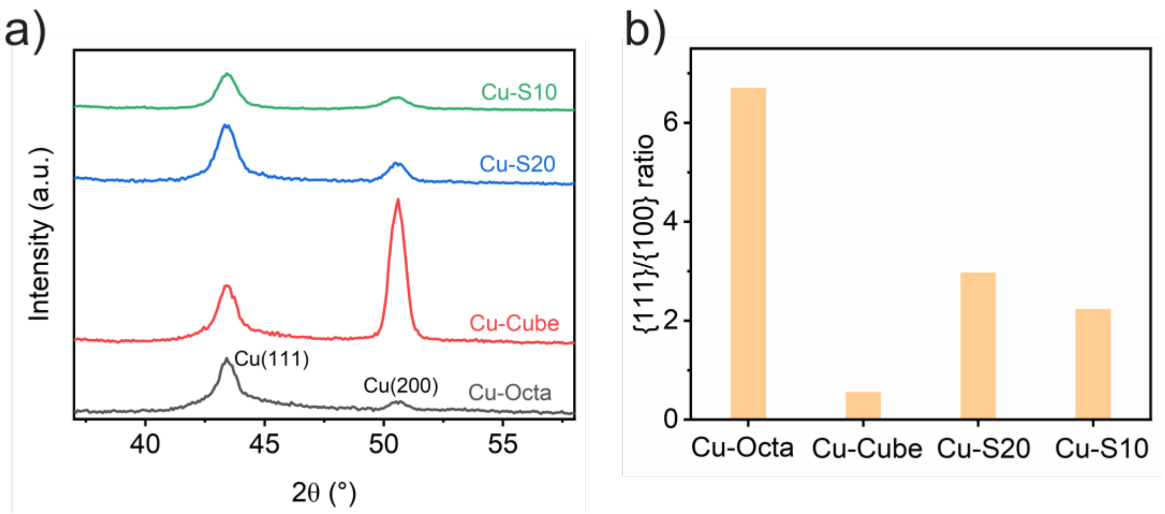

Figure S18. (a) IPGID patterns and (b) extracted {111}/{100} facet ratio of catalysts after NO_3RR at -0.4 V_{RHE}. The {111}/{100} facet ratios were calculated by dividing the area of Cu(111) diffraction peaks by that of Cu(200) diffraction peaks from IPGID patterns.

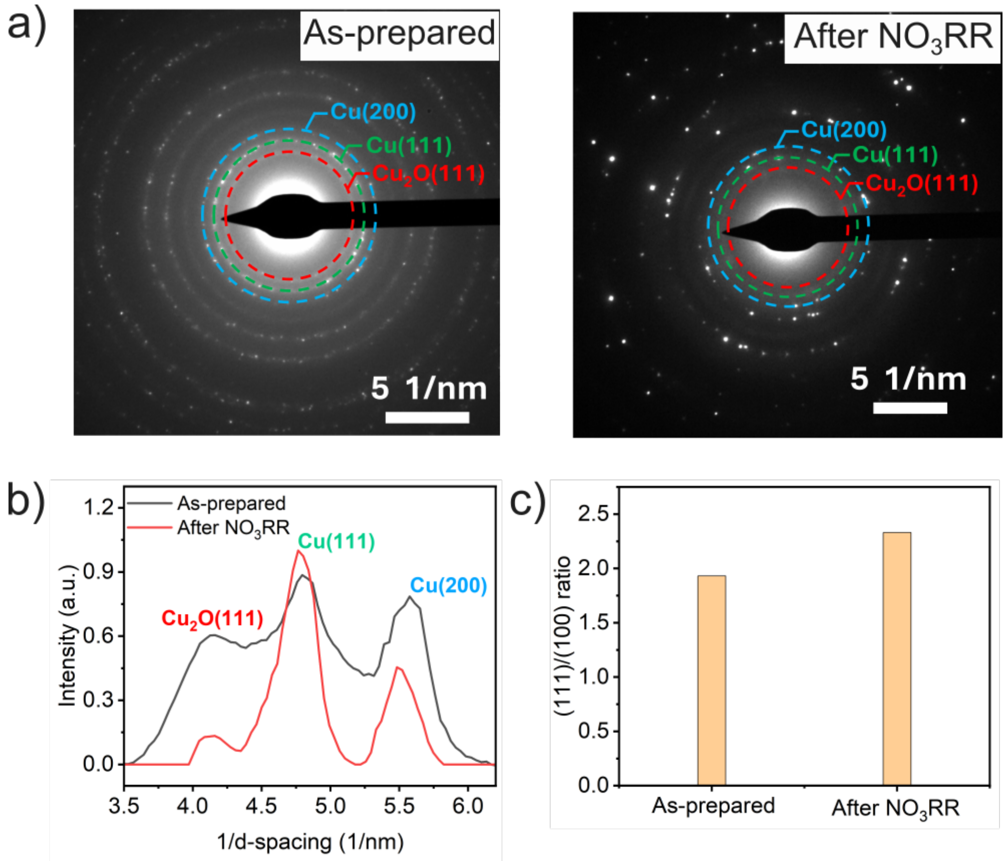

Figure S19. (a) ED patterns of Cu-Octa + Cu-Cube mixed sample of as prepared and after NO_3RR (corresponding to TEM images shown in Figure 4a), (b) corresponding ED integrated

intensity profiles and (c) comparison of Cu{111}/Cu{100} facet ratio. The physically mixed sample was prepared by mixing the catalyst ink of Cu-Octa and Cu-Cube with specific concentration to achieve the {111}/{100} facet ratio to 2. The presence of Cu_2O in the samples can be caused by unavoidable exposure to air.

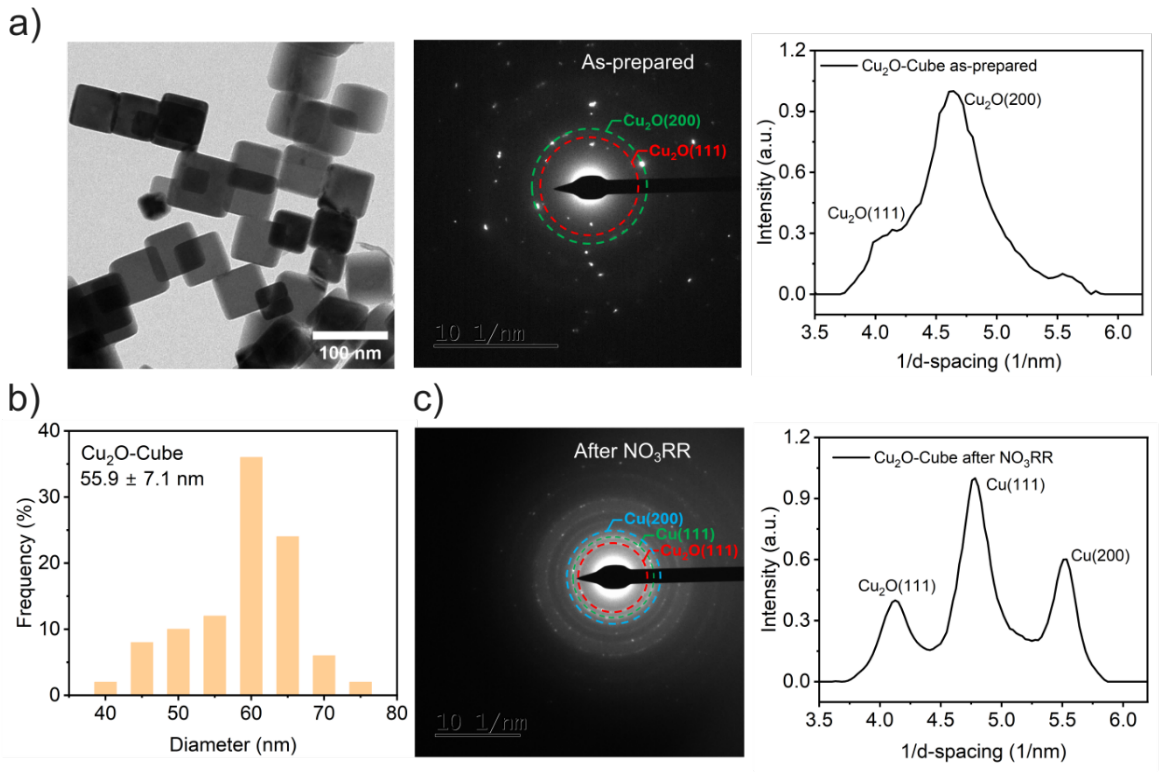

Figure S20. (a) Representative bright-field TEM image, ED patterns and ED integrated intensity profiles of as-prepared Cu_2O-Cube, (b) particle size distribution of as-prepared Cu_2O-Cube (b) and ED patterns and (c) ED integrated intensity profiles of post-NO_3RR Cu_2O-Cube at -0.4 V_{RHE}.

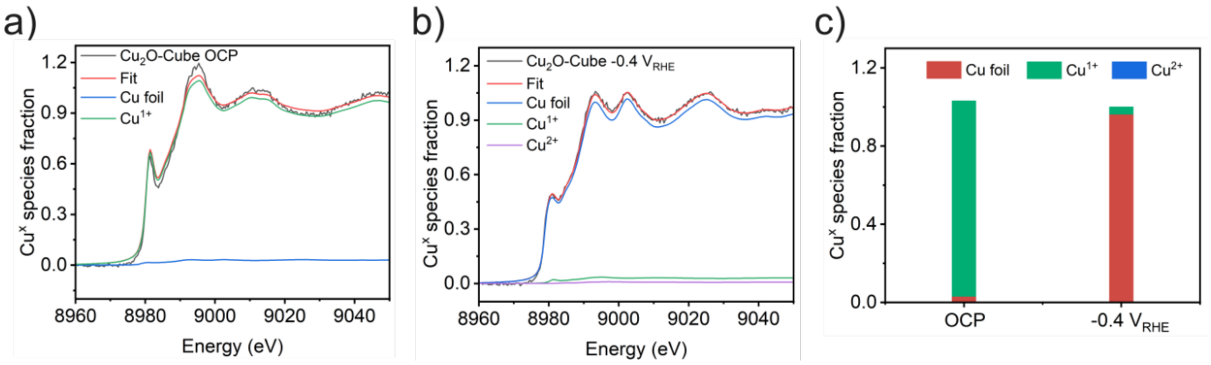

Figure S21. (a,b) XANES spectra together with the fitted spectra using Model 4 (Cu, Cu_2O, CuO) for Cu_2O-Cube measured at OCP (a) and at -0.4 V_{RHE} (b). (c) LCA of Cu_2O-Cube displaying Cu^x species fraction acquired operando at OCP and at -0.4 V_{RHE} .

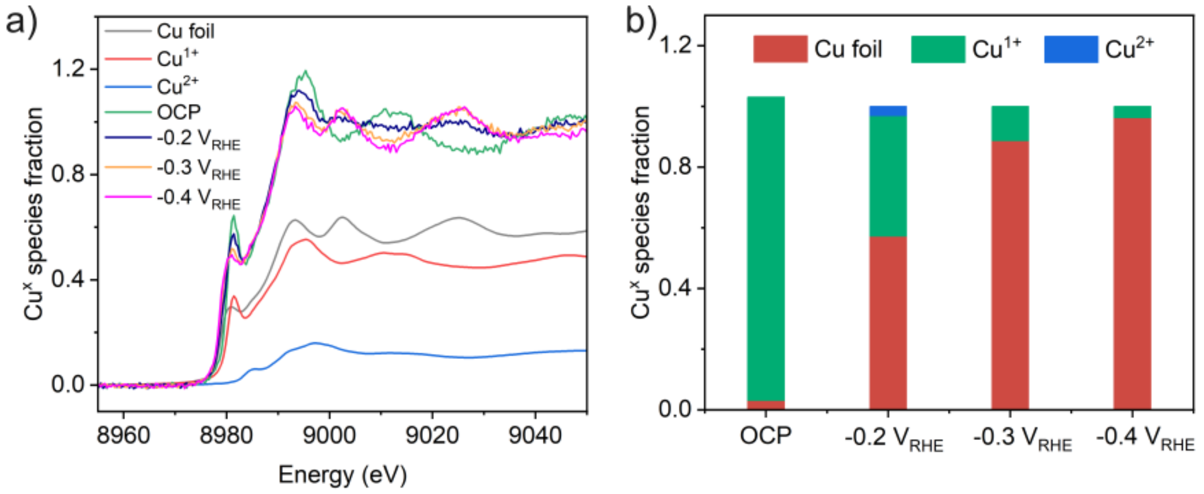

Figure S22. (a) XANES spectra and (b) linear combination analysis of Cu_2O-Cube acquired during NO_3RR measurements.

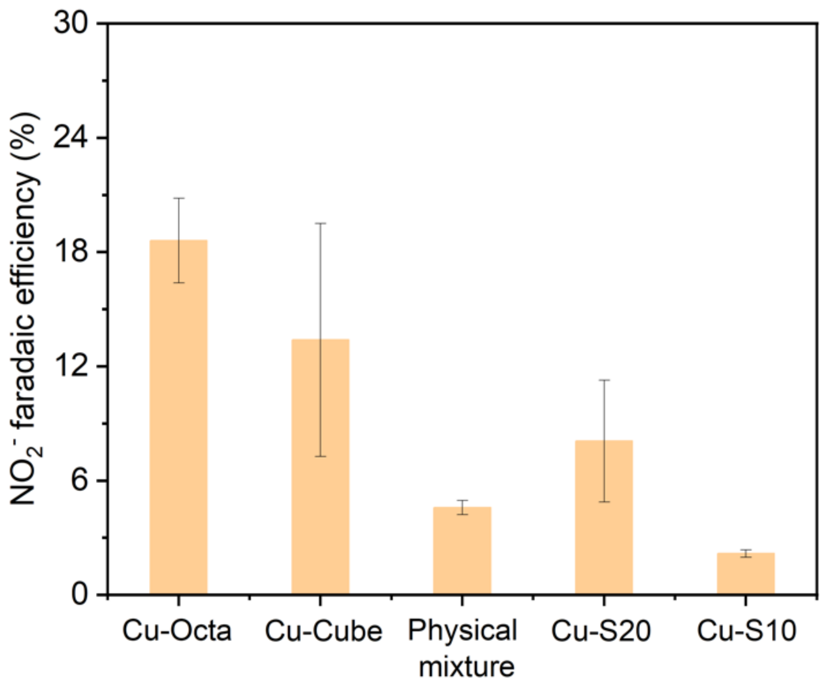

Figure S23. Faradaic efficiency for NO_2^- during NO_3RR at -0.4 V_{RHE} for Cu-Octa, Cu-Cube, physical mixture, Cu-S20 and Cu-S10.

The FE for NO_2^- serves as an indicator for the extent to which the NO_2^- intermediate can be efficiently converted (i.e. lower FE, more efficient conversion). The data indicate that the NO_2^- intermediate is more efficiently consumed in the subsequent reduction step by the catalyst exhibiting on their surface both {111} and {100} surfaces, specifically the physical mixture and the Cu spheres, with the Cu-S10 exhibiting the lowest FE value, indicating the most efficient conversion of NO_2^-.

References

(1) Strach, M.; Mantella, V.; Pankhurst, J. R.; Iyengar, P.; Loiudice, A.; Das, S.; Corminboeuf, C.; Van Beek, W.; Buonsanti, R. Insights into Reaction Intermediates to Predict Synthetic Pathways for Shape-Controlled Metal Nanocrystals. *J Am Chem Soc* **2019**, *141* (41), 16312–16322. https://doi.org/10.1021/jacs.9b06267.

(2) Mantella, V.; Strach, M.; Frank, K.; Pankhurst, J. R.; Stoian, D.; Gadiyar, C.; Nickel, B.; Buonsanti, R. Polymer Lamellae as Reaction Intermediates in the Formation of Copper Nanospheres as Evidenced by In Situ X-Ray Studies. *Angewandte Chemie - International Edition* **2020**, *59* (28), 11627–11633. https://doi.org/10.1002/anie.202004081.

(3) Suen, N.-T.; Kong, Z.-R.; Hsu, C.-S.; Chen, H.-C.; Tung, C.-W.; Lu, Y.-R.; Dong, C.-L.; Shen, C.-C.; Chung, J.-C.; Chen, H. M. Morphology Manipulation of Copper Nanocrystals and Product Selectivity in the Electrocatalytic Reduction of Carbon Dioxide. *ACS Catal* **2019**, *9* (6), 5217–5222. https://doi.org/10.1021/acscatal.9b00790.

(4) Iyengar, P.; Kolb, M. J.; Pankhurst, J. R.; Calle-Vallejo, F.; Buonsanti, R. Elucidating the Facet-Dependent Selectivity for CO2 Electroreduction to Ethanol of Cu–Ag Tandem Catalysts. *ACS Catal* **2021**, *11* (8), 4456–4463. https://doi.org/10.1021/acscatal.1c00420.

(5) Bai, L.; Franco, F.; Timoshenko, J.; Rettenmaier, C.; Scholten, F.; Jeon, H. S.; Yoon, A.; Rüscher, M.; Herzog, A.; Haase, F. T.; Kühl, S.; Chee, S. W.; Bergmann, A.; Beatriz, R. C. Electrocatalytic Nitrate and Nitrite Reduction toward Ammonia Using Cu2O Nanocubes: Active Species and Reaction Mechanisms. *J Am Chem Soc* **2024**, *146* (14), 9665–9678. https://doi.org/10.1021/jacs.3c13288.

(6) Klinger, M.; Jäger, A. Crystallographic Tool Box (CrysTBox): Automated Tools for Transmission Electron Microscopists and Crystallographers. *J Appl Crystallogr* **2015**, *48*, 2012–2018. https://doi.org/10.1107/S1600576715017252.

(7) Wang, M.; Nikolaou, V.; Loiudice, A.; Sharp, I. D.; Llobet, A.; Buonsanti, R. Tandem Electrocatalytic CO2 Reduction with Fe-Porphyrins and Cu Nanocubes Enhances Ethylene Production. *Chem Sci* **2022**, *13* (43), 12673–12680. https://doi.org/10.1039/D2SC04794B.

(8) Wei, M.; Li, S.; Wang, X.; Zuo, G.; Wang, H.; Meng, X.; Wang, J. A Perspective on Cu-Based Electrocatalysts for Nitrate Reduction for Ammonia Synthesis. *Advanced Energy and Sustainability Research* **2024**, *5*, 2300173. https://doi.org/10.1002/aesr.202300173.

(9) Fu, X.; Zhao, X.; Hu, X.; He, K.; Yu, Y.; Li, T.; Tu, Q.; Qian, X.; Yue, Q.; Wasielewski, M. R.; Kang, Y. Alternative Route for Electrochemical Ammonia Synthesis by Reduction of Nitrate on Copper Nanosheets. *Appl Mater Today* **2020**, *19*, 100620. https://doi.org/10.1016/j.apmt.2020.100620.

(10) Fu, Y.; Wang, S.; Wang, Y.; Wei, P.; Shao, J.; Liu, T.; Wang, G.; Bao, X. Enhancing Electrochemical Nitrate Reduction to Ammonia over Cu Nanosheets via Facet Tandem Catalysis. *Angewandte Chemie - International Edition* **2023**, *62*, e202303327. https://doi.org/10.1002/anie.202303327.

(11) Hu, Q.; Qin, Y.; Wang, X.; Wang, Z.; Huang, X.; Zheng, H.; Gao, K.; Yang, H.; Zhang, P.; Shao, M.; He, C. Reaction Intermediate-Mediated Electrocatalyst Synthesis Favors Specified Facet and Defect Exposure for Efficient Nitrate-Ammonia Conversion. *Energy Environ Sci* **2021**, *14* (9), 4989–4997. https://doi.org/10.1039/d1ee01731d.

(12) Geng, J.; Ji, S.; Xu, H.; Zhao, C.; Zhang, S.; Zhang, H. Electrochemical Reduction of Nitrate to Ammonia in a Fluidized Electrocatalysis System with Oxygen Vacancy-Rich CuO: Xnanoparticles. *Inorg Chem Front* **2021**, *8* (24), 5209–5213. https://doi.org/10.1039/d1qi01062j.

(13) Wu, K.; Sun, C.; Wang, Z.; Song, Q.; Bai, X.; Yu, X.; Li, Q.; Wang, Z.; Zhang, H.; Zhang, J.; Tong, X.; Liang, Y.; Khosla, A.; Zhao, Z. Surface Reconstruction on Uniform Cu Nanodisks Boosted Electrochemical Nitrate Reduction to Ammonia. *ACS Mater Lett* **2022**, *4* (4), 650–656. https://doi.org/10.1021/acsmaterialslett.2c00149.

(14) Zhao, X.; Jia, X.; He, Y.; Zhang, H.; Zhou, X.; Zhang, H.; Zhang, S.; Dong, Y.; Hu, X.; Kuklin, A. V.; Baryshnikov, G. V.; Ågren, H.; Hu, G. Two-Dimensional BCN Matrix Inlaid with Single-Atom-Cu Driven Electrochemical Nitrate Reduction Reaction to Achieve Sustainable Industrial-Grade Production of Ammonia. *Appl Mater Today* **2021**, *25*. https://doi.org/10.1016/j.apmt.2021.101206.

(15) Yang, J.; Qi, H.; Li, A.; Liu, X.; Yang, X.; Zhang, S.; Zhao, Q.; Jiang, Q.; Su, Y.; Zhang, L.; Li, J. F.; Tian, Z. Q.; Liu, W.; Wang, A.; Zhang, T. Potential-Driven Restructuring of Cu Single Atoms to Nanoparticles for Boosting the Electrochemical Reduction of Nitrate to Ammonia. *J Am Chem Soc* **2022**, *144* (27), 12062–12071. https://doi.org/10.1021/jacs.2c02262.

(16) Nösberger, S.; Du, J.; Quinson, J.; Berner, E.; Zana, A.; Wiberg, G. K. H.; Arenz, M. The Gas Diffusion Electrode Setup as a Testing Platform for Evaluating Fuel Cell Catalysts: A Comparative RDE-GDE Study. *Electrochemical Science Advances* **2023**, *3* (1), e2100190. https://doi.org/https://doi.org/10.1002/elsa.202100190.

(17) Wang, M.; Loiudice, A.; Okatenko, V.; Sharp, I. D.; Buonsanti, R. The Spatial Distribution of Cobalt Phthalocyanine and Copper Nanocubes Controls the Selectivity towards C2 Products in Tandem Electrocatalytic CO2 Reduction. *Chem Sci* **2023**, *14* (5), 1097–1104. https://doi.org/10.1039/d2sc06359j.

(18) Zhan, C.; Dattila, F.; Rettenmaier, C.; Herzog, A.; Herran, M.; Wagner, T.; Scholten, F.; Bergmann, A.; López, N.; Roldan Cuenya, B. Key Intermediates and Cu Active Sites for CO2 Electroreduction to Ethylene and Ethanol. *Nat Energy* **2024**, *9* (12), 1485–1496. https://doi.org/10.1038/s41560-024-01633-4.

(19) Yang, Y.; Louisia, S.; Yu, S.; Jin, J.; Roh, I.; Chen, C.; Fonseca Guzman, M. V.; Feijóo, J.; Chen, P. C.; Wang, H.; Pollock, C. J.; Huang, X.; Shao, Y. T.; Wang, C.; Muller, D. A.; Abruña, H. D.; Yang, P. Operando Studies Reveal Active Cu Nanograins for CO2 Electroreduction. *Nature* **2023**, *614* (7947), 262–269. https://doi.org/10.1038/s41586-022-05540-0.

(20) Zhang, J.; Huang, L.; Tjiu, W. W.; Wu, C.; Zhang, M.; Bin Dolmanan, S.; Wang, S.; Wang, M.; Xi, S.; Aabdin, Z.; Lum, Y. Evidence for Distinct Active Sites on Oxide-Derived Cu for Electrochemical Nitrate Reduction. *J Am Chem Soc* **2024,** 146, 30708–30714. https://doi.org/10.1021/jacs.4c13219.
